# Exploiting Phenylpropanoid Derivatives to Enhance the Nutraceutical Values of Cereals and Legumes

**DOI:** 10.3389/fpls.2016.00763

**Published:** 2016-06-03

**Authors:** Sangam L. Dwivedi, Hari D. Upadhyaya, Ill-Min Chung, Pasquale De Vita, Silverio García-Lara, Daniel Guajardo-Flores, Janet A. Gutiérrez-Uribe, Sergio O. Serna-Saldívar, Govindasamy Rajakumar, Kanwar L. Sahrawat, Jagdish Kumar, Rodomiro Ortiz

**Affiliations:** ^1^International Crops Research Institute for the Semi-Arid TropicsPatancheru, India; ^2^Department of Agronomy, Kansas State UniversityManhattan, KS, USA; ^3^UWA Institute of Agriculture, University of Western AustraliaCrawley, WA, Australia; ^4^Department of Applied Life Science, College of Life and Environmental Science, Konkuk UniversitySeoul, Korea; ^5^Consiglio per la Ricerca in Agricoltura e l'Analisi dell'Economia Agraria, Centro di Ricerca per la CerealicolturaFoggia, Italy; ^6^Tecnológico de Monterrey, Centro de Biotecnología-FEMSA, Escuela de Ingeniería y CienciasMonterrey, Mexico; ^7^Sevita InternationalInkerman, ON, Canada; ^8^Swedish University of Agricultural SciencesAlnarp, Sweden

**Keywords:** anthocyanins, cereals, flavonoids, *Genotype* × *Environment* interaction, genetics and biosynthesis, germplasm, legumes, phenolics

## Abstract

Phenylpropanoids are a diverse chemical class with immense health benefits that are biosynthesized from the aromatic amino acid L-phenylalanine. This article reviews the progress for accessing variation in phenylpropanoids in germplasm collections, the genetic and molecular basis of phenylpropanoid biosynthesis, and the development of cultivars dense in seed-phenylpropanoids. Progress is also reviewed on high-throughput assays, factors that influence phenylpropanoids, the site of phenylpropanoids accumulation in seed, *Genotype* × *Environment* interactions, and on consumer attitudes for the acceptance of staple foods rich in phenylpropanoids. A paradigm shift was noted in barley, maize, rice, sorghum, soybean, and wheat, wherein cultivars rich in phenylpropanoids are grown in Europe and North and Central America. Studies have highlighted some biological constraints that need to be addressed for development of high-yielding cultivars that are rich in phenylpropanoids. Genomics-assisted breeding is expected to facilitate rapid introgression into improved genetic backgrounds by minimizing linkage drag. More research is needed to systematically characterize germplasm pools for assessing variation to support crop genetic enhancement, and assess consumer attitudes to foods rich in phenylpropanoids.

## Introduction

Polyphenols are secondary metabolites that are synthesized by plants from the amino acid phenylalanine. They are derived from the C_6_-C_3_ (phenyl-propane) skeleton. Plant biosynthesis produces various phenols that can be grouped generally as flavonoids and phenolics. Flavones, flavonols, flavanones, flavan-3-ols, anthocyanidins, isoflavones, coumarins, stilbenes, and lignans are the main flavonoids (Pereira et al., 2009). These are structurally distinct because of their specific hydroxylation, methylation, and conjugation patterns, with various monosaccharides and disaccharides (Graf et al., 2005; He and Giusti, 2010; Ignat et al., 2011; de Oliveira et al., 2014). Some flavonoids are exclusively synthesized by specific plants, such as phlobaphenes in maize (Sharma et al., 2012) and isoflavonoids in legumes (Mazur et al., 1998). Phenolic acids exist primarily as benzoic-acid and cinnamic-acid derivatives, and can occur in the free or conjugated forms. Gallic, *p*-benzoic, protocatechuic, syringic, and vanillic acids are benzoic-acid derivatives, while caffeic, ferulic, *p*-coumaric, and sinapic acids are cinnamic-acid derivatives (Razzaghi-As et al., 2013; de Oliveira et al., 2014).

Polyphenols are involved in various plant functions. They provide shades of color to flowers (color attracts pollinators), fruit, vegetables, and grains. Their bitter or astringent taste attributes can repel birds and other animals. Polyphenols protect plants from UV radiation and provide defense against environmental stress, as well as against pathogens and pests, and they act as signaling molecules to facilitate symbiotic nitrogen fixation and confer seed dormancy (Subramanian et al., 2007; Yang et al., 2008; Gu et al., 2011; Soares et al., 2013; de Oliveira et al., 2014). The phenylpropanoids have received considerable attention owing to their potential benefits for human health.

This article reviews the phenylpropanoid constituents, with emphasis on the mining of germplasm variation for flavonoids (excluding lignans and stilbenes) and phenolics, the site and factors that influence phenylpropanoid accumulation in seed, the *Environment, Genotype*, and *Genotype* × *Environment* interactions, the genetic and molecular basis of phenylpropanoid biosynthesis, and the development of seed-phenylpropanoid dense cultivars. Updates on flavonoids as promoters of micronutrient bioavailability and human health, high-throughput assays for estimation of phenylpropanoids, and consumer attitudes to accepting phenylpropanoid-dense foods are also included here.

## Human health and micronutrient bioavailability

As a dietary component, phenylpropanoids have health-promoting properties due to their high antioxidant capacity that has been shown in both *in-vivo* and *in*-*vitro* systems (Cook and Samman, 1996). The antioxidant capacity of flavonoids is conferred by the high number of hydroxyl substitutions in each flavonoid molecule, which has a direct effect on the donating ability of hydrogen atoms to scavenge free radicals (Pietta, 2000). The food matrix and its processing conditions have strong effects on the retention of these compounds and their use as a functional food ingredient (Chavez-Santoscoy et al., 2016). Unfortunately, the availability of micronutrients and phytochemicals has been studied through reductionist and pharmacological approaches to date, while the beneficial effects of health-promoting compounds should in addition be analyzed holistically as food is not a drug. Furthermore, a compound that has been shown to be bioactive might have different effects when tested in a complex matrix (Fardet and Rock, 2014). Hence, it is important to identify and validate the stability of bioactive compounds both in isolation as well as once they have been incorporated into functional foods.

Combining cereals and legumes improves not only the nutritional content of these foods, but also their health-promoting effects. Anton et al. (2008) investigated the effects of red, black, and pinto or navy common bean (*Phaseolus vulgaris* L.) flour in wheat tortilla, and noted that tortillas with black beans had higher levels of crude protein, total phenols, and *in-vitro* antioxidant activity than those solely made with wheat flour. Black bean flavonoids, such as quercetin-3-*O*-glucoside, can have strong effects on down-regulation of expression of lipogenic proteins (Chavez-Santoscoy et al., 2014; Ramírez-Jiménez et al., 2015). Anthocyanin consumption from the original food matrix or once extracted inhibits tumorigenesis of esophageal cancer and changes inflammatory markers in rats (Peiffer et al., 2016). For example, 3-*O*-glucosylated anthocyanins (i.e., delphinidin, petunidin, malvidin) in the seed-coat extract of black violet beans is associated with antioxidant and anti-inflammatory activities (Oomah et al., 2010; Mojica et al., 2015). Furthermore, black-seeded common bean cultivars are superior to other food crops as nutraceutical supplements (Chavez-Santoscoy et al., 2013; Guajardo-Flores et al., 2015; Rosales-Serna et al., 2015).

Non-communicable diseases such as cancer, diabetes, heart disease, and stroke are some of the major challenges to global health, and they are often associated with the negative effects of globalization, rapid urbanization, diet, and increasingly sedentary lifestyles (Wagner and Brath, 2012). Flavonoids are implicated in the prevention of cardiovascular diseases (Mink et al., 2007; Curtis et al., 2009; Weseler et al., 2011). Soluble vascular adhesion molecule-1 (sVCAM-1) is an important biomarker that is used to predict the risk of death from coronary heart diseases (Blankenberg et al., 2001). Phenolic metabolites have stronger effects on reducing the sVCAM-1 levels than the corresponding flavonoids (Warner et al., 2016). Thus, the metabolism of flavonoids is critical to increases in their vascular efficacy, and this explains the differences among individuals when phenolic compounds are tested *in-vivo*. Colon cancer is a major public health burden in both developed and developing countries (Torre et al., 2015). Adoption of a Western diet is the major cause of colon cancer (Center et al., 2009). Sorghum [*Sorghum bicolor* (L.) Conrad Moench] flavonoids contribute to colon cancer prevention at concentrations that are achievable through the diet (Yang et al., 2014). They also noted that the composition of phenolic compounds, not content, has a major effect on estrogenic activity and on the protective efficacy of sorghum in preventing colon cancer.

Another global challenge to human health is being overweight or obese (Ng et al., 2014). Increased consumption of flavonoid-rich fruit and vegetables can help with weight management. Higher intake of foods rich in flavonols, flavan-3-ols, anthocyanins, and flavonoid polymers has been associated with less weight gain among men and women aged 27–65 years who were followed for up to 24 years (Bertoia et al., 2015). This association remained statistically significant for anthocyanins after further adjustment for fiber intake, which indicated that food sources with a high contents of anthocyanin and flavonoid polymers can be associated with less weight gain through mechanisms other than fiber content.

Phytic acid is the major contributor to reduced bioavailability of micronutrients in cereals and legumes, while polyphenols are major inhibitors of iron (Fe) absorption and act in a manner similar to phytate, by complexing Fe (Dwivedi et al., 2012). The metal-chelating characteristics of flavonoids are an important factor in antioxidant activities (Bonina et al., 1996; Boyle et al., 2000). Studies have demonstrated high binding capacities of polyphenols for Fe (Teucher et al., 2004; Perron and Brumaghim, 2009; Cercamondi et al., 2014). Flavonoids can bind nonheme iron and inhibit intestinal absorption of Fe from food (Mladenka et al., 2011; Corcoran et al., 2012). Nonheme iron is the principal form of Fe in plant foods, dairy products, and iron supplements. Flavonoids in colored bean seed coats strongly inhibit Fe bioavailability in bean digests (Hu et al., 2006). The chelation of metal ions by flavonoids can render the ions inactive in the generation of radicals, or alternately, flavonoids can themselves intercept radicals that are generated (Tako et al., 2015). Some polyphenols can reduce Fe(III) to Fe(II) (Perron and Brumaghim, 2009), thus promoting iron bioavailability. Catechin, 3,4-dihydroxybenzoic acid, kaempferol, and kaempferol 3-glucoside have been shown to promote Fe uptake, while myricetin, myricetin 3-glucoside, quercetin, and quercetin 3-glucoside inhibit Fe uptake (Hart et al., 2015). These inhibitors are, however, found in greater amounts than the promoters, which is consistent with the net inhibitory effects observed for black bean seed coats. The promotion of Fe uptake by some polyphenols and the identification of specific polyphenols that inhibit Fe uptake suggest the potential for breeding beans with improved Fe nutritional quality (Grieger et al., 2008; Tako and Glahn, 2010; Tako et al., 2015).

Flavonoids enhance the function of vitamin C, thus improving its absorption and protecting it from oxidation (Pietta, 2000). Vitamin C is a multi-functional micronutrient that is required in its reduced form (L-ascorbic acid) for many enzymatic reactions, and as a scavenger of free radicals generated from numerous physiological and biochemical processes (Evans and Halliwell, 2001). Flavonoids might also regenerate other antioxidants, such as tocopherols, by donating a hydrogen atom to the tocopheroxyl radical in a way that is reminiscent of their action on vitamin C (Boyle et al., 2000).

## Analytical determination of phenylpropanoids

Various assays for determining phenolic compounds have been developed (Bravo, 1998; Khoddami et al., 2013). These can be classified as those that quantify the total phenolics content, or those that quantify or identify a specific group or class of phenolics. Colorimetric methods are used to determine the total phenolics levels, and high performance liquid chromatography (HPLC) is used to identify and quantify specific phenolic compounds. The colorimetric methods include Folin-Ciocalteu assays (Singleton et al., 1999), Prussian blue tests (Graham, 1992), ferric ammonium citrate tests (International Organization for Standardization), and vanillin-HCl and butanol-HCl tests (Price et al., 1978; Watterson and Butler, 1983; Porter et al., 1986). These methods have been used for total phenol determination in barley (*Hordeum vulgare* L.), common bean (Hart et al., 2015), rice (*Oryza sativa* L.; Begum et al., 2015), sorghum (Beta et al., 1999; Waniska and Rooney, 2000; Dykes et al., 2005; Dlamini et al., 2007; Chiremba et al., 2012), soybean [*Glycine max* (L.) Merr.; Nikolova et al., 2014; Phommalath et al., 2014], einkorn wheat (*Triticum monococcum* L.), and bread wheat (*Triticum aestivum* L.; Fogarasi et al., 2015). HPLC techniques coupled with photodiode array, fluorescence, or mass spectroscopy detectors have been used to identify and quantify specific phenolics in rice (Zhou et al., 2004), sorghum (Svensson et al., 2010; Chiremba et al., 2012), soybean (Kim et al., 2014; Kumar et al., 2015), and wheat (Ficco et al., 2014). Sriseadka et al. (2012) identified and quantified 11 flavonoids in black rice using liquid chromatography electrospray ionization tandem mass spectrometry, six of which were reported for the first time. Due to their chemical nature, the extraction method used, the standards used, and the presence of interfering substances, the various methods available for the analysis of phenylpropanoids remain too complex, time consuming, and labor intensive for routine screening. These limitations represent a bottleneck for modern studies of functional genomics and modern plant breeding (Furbank and Tester, 2011).

Near infrared (NIR) spectroscopy appears to be an appropriate technique to achieve these goals. It is faster than chromatographic or wet-chemical methods, and it can provide correct identification in less than 2 min without destroying the sample. NIR spectroscopy simultaneously measures several quality traits that are routinely tested in cereals (Bao et al., 2001, 2007; Wu et al., 2002; Wu and Shi, 2004, 2007; Osborne, 2006). It has been applied for the determination of phenolic compounds, flavonoid content, and antioxidant capacity in food derived from rice, sorghum, cocoa (*Theobroma cacao* L.), wine, grapes (*Vitis vinifera* L.), apples (*Malus domestica* Borkh., 1803), and tea [*Camellia sinensis* (L.) Kuntze; Whitacre et al., 2003; Cozzolino et al., 2004, 2008; Janik et al., 2007; Chen et al., 2008; Zhang et al., 2008; Pissard et al., 2013; Dykes et al., 2014; Hassan et al., 2015]. NIR spectroscopy has a high degree of precision when applied to the analysis of nutraceutical and antioxidant compounds in terms of their concentrations and antioxidant activities in foods (Ignat et al., 2011; Bunaciu et al., 2012; Lu and Rasco, 2012; Bittner et al., 2013; Cozzolino, 2015). Hence, NIR spectroscopy can provide very useful qualitative and quantitative information on different antioxidants, combined with its simplicity and low cost. Calibration development is critical to establishing a successful method based on NIR spectroscopy. Although the polyphenol quantification method is well established, a modified Folin-Cioalteu method incorporates the convenience of spectrometric measurements using 96-well microplates (Zhang et al., 2006). Automation of the workflow using 384-well microplates, as optimized for robotics, automated readers, and liquid handling systems, makes it possible to use much smaller quantities of reagents and solvents, and to significantly increase the throughput of the analysis of the compounds tested.

A 96-well microtiter assay that has been used for decades in the pharmaceutical industry has been standardized for screening total phenolic, flavonoid, and tannin contents, and for 2,2-diphenyl-1-picrylhydrazyl free radical (DPPH)-scavenging activity in grape, sorghum (Herald et al., 2012, 2014; Bobo-García et al., 2015) and wheat (Cheng et al., 2006) extracts. This assay is thus as robust and reproducible as the conventional method for determining phenolic compounds. Most commonly used assays for measuring antioxidant activity, including those with DPPH, have both conceptual and technical limitations (Apak et al., 2013; Tian and Schaich, 2013; Xie and Schaich, 2014; Schaich et al., 2015), especially when comparing different food matrices. Although, it is necessary to continue investigating antioxidant efficacy using fundamental chemistry (Schaich et al., 2015), high-throughput assays remain a strategic asset to monitor variations in antioxidant activity in large germplasm collections. Laus et al. (2015) proposed the QUENCHER_ABTS_ for determination of antioxidant capacity, which is quick, easy, new, cheap, and reproducible. This method can also be used to accurately discriminate antioxidant capacity associated with the insoluble-bound phenolics of wheat grain without any preliminary sample extraction. Thus, it allows good discrimination among wheat genotypes, with better physiological significance than the classical Trolox equivalent antioxidant capacity and 2,2′-azinobis-(3-ethylbenzothiazoline-6-sulfonic acid) measurements. In addition, high-throughput oxygen-radical absorbance capacity assays conducted with more detailed and revised protocols might be a valuable alternative to the common testing methods for antioxidants (Huang et al., 2002).

Imaging systems working in the UV, visible, NIR, and Raman spectral ranges of the electromagnetic spectrum can be used to obtain information on composition and distribution of phenylpropanoids. As the hyperspectral imaging techniques combine spectroscopic and imaging systems, they can be used for detecting very low levels of chemical constituents in cereal and legume grain along with spatial distributions (Budevska, 2002; Kezhu et al., 2014; Mahajan et al., 2015). The hyperspectral microspectroscopic imaging techniques have been used to study the endosperm/aleurone/pericarp area of mature kernels of maize (*Zea mays* L.; Budevska, 2002). Hyperspectral imaging systems have also been used to develop single kernel methods to determine the physical and biochemical traits of cereals (Codgill et al., 2002, 2004; Fox and Manley, 2014). These methods focus on the calibration of the hyperspectral imaging instrument to predict the constituent concentrations in single kernels using NIR hyperspectral images.

## Environment and genotype effects on phenylpropanoids

The flavonoid content is dominantly influenced by both genotype and environment. Better understanding of genotype and environment effects is a prerequisite to selecting food crops with enhanced flavonoids, so that cultivars high in flavonoids can be targeted to suitable environments.

Most studies on legumes as a source of functional foods has focused on soybean. For example, field research on six non-transgenic soybean genotypes grown in 23 environments (E) was carried out in Argentina to study seed nutraceutical composition. This showed that although *Environment* was the most important source, *Genotype* and *Genotype* × *Environment* interactions also had significant effects on grain nutraceutical composition (Carrera et al., 2014). These results agreed with those reported earlier by Lee et al. (2003), who showed that in South Korea, the main effects of *Year, Site, Genotype*, and all possible interactions between these were significant for all of the isoflavones. Murphy et al. (2009a) reported that breeding for relative isoflavone content was possible in two soybean populations in Ontario, Canada. Nonetheless, they cautioned that breeding for absolute stability is a challenge, because of the very strong effects of the environment on isoflavone accumulation in soybean. Temperature, precipitation, and soil moisture in the field conditions are the most important factors that influence flavonoid contents in soybean genotypes (Kim et al., 2012a), while temperature and soil moisture status change the isoflavone and anthocyanin contents of soybean under controlled conditions (Caldwell et al., 2005; Lozovaya et al., 2005; Chennupati et al., 2011).

Variable effects of genotype and environment on phenylpropanoid compounds were reported in wheat. Mpofu et al. (2006) and Fernandez-Orozco et al. (2010) showed greater contribution of environment than genotypes on flavonoid and phenolic content, while others reported greater genotypic effects than environment on polyphenols (Martini et al., 2015; Rascio et al., 2015). Variation in sowing date is also reported to cause significant differences in polyphenol content; i.e., polyphenols were increased in spring-sown compared to winter-sown wheats (Rascio et al., 2015). This variation depended on genotypes. A negative effect of spring sowing on grain yield was observed, but positive effects on 1000-kernel weight suggested that high temperatures can lead to a net accumulation of healthy substances in grain, but not a relative increase due to grain shriveling. There is a need to confirm these data in multiple environments, because such an approach facilitates the specific enrichment of cereal-based foods as per consumer requirements (Rascio et al., 2015). Total phenolics and phenolic acids were mostly affected by the environment in a 3-year field evaluation of durum wheat in Italy (Martini et al., 2015).

Changes in anthocyanin content of wheat cultivars were associated with sink-source (i.e., availability of carbohydrate for anthocyanin production) transition, grain position (i.e., the anthocyanin content decreased when grain position was more distal), and physiological stage of the crop. Magnesium fertilization and early harvest (at physiological maturity) increased anthocyanin content and concentrations by 65 and 39%, respectively (Bustos et al., 2012). Heat stress can adversely affect compounds that are beneficial or detrimental to human health (Dias and Lidon, 2010; Laino et al., 2010). More recently, de Leonardis et al. (2015) reported that in addition to affecting seed nutritional composition, 5-day heat stress (37°C) after flowering impacted on the antioxidant capacities and metabolic profiles of durum wheats. This response to heat stress was genotype-dependent, with most analyzed metabolites increasing in “Primadur” (high in seed carotenoids), but decreasing in “T1303” (high in seed anthocyanin).

Goufo and Trindade (2014) indicated that among four types of rice that were ranked by color, black rice cultivars were the highest in flavonoids, followed by the purple, red, and brown cultivars. These results were influenced by both the genotype and the environment. For example, elevated carbon dioxide reduced total phenolics, total flavonoids, and individual flavonoids (flavone, and some unidentified flavonoids) in rice kernels and all of the rice milling fractions. These results emphasize the importance of future atmospheric scenarios in breeding rice cultivars with increased antioxidant content (Goufo and Trindade, 2014; Goufo et al., 2014). The distribution of flavonoids in rice cultivars was not significantly affected by agronomic practices, but flavonoid content was significantly affected by the season, and the genotype, and by their interactions (de Mira et al., 2009; Liu et al., 2013).

Taleon et al. (2012) investigated the effects of both the genotype and environment on flavonoid concentrations in black sorghum grain in Texas. Significant variation due to the genotype, the environment, and their interactions was observed. Most of the variation was, however, associated with *Genotype* or *Environment*. The genotypic variation was greater than that for environment variation for flavones, while for flavanones, the environment variation was greater. Hence, the identification of both the best genotype and environment will provide the highest yields of total flavonoid content, and sorghum breeders need to evaluate these traits in multiple environments to select the genotypes with stable and high content of flavonoids of interest (Taleon et al., 2012). Similar results were reported and conclusions drawn for flavonoid content in red and lemon-yellow sorghum grain. The evaluation of genotypes in multiple environments was emphasized to obtain the best data related to the flavonoid content (Taleon et al., 2014).

Functional components including starch, protein, dietary fiber, and phenolic antioxidants were considerably influenced by the environment, genotype, type (i.e., hull-less, with hulls) and their interactions for barley grown in 23 different environments in eastern Canada (Abdel-Aal and Choo, 2014). The starch, which is the main available carbohydrate in barley, varied according to year, barley type and individual cultivars or lines. The absence of hulls tended to enhance the protein and total antioxidant capacity (Abdel-Aal and Choo, 2014).

Clearly, *Environment, Genotype*, and *Genotype* × *Environment* interactions have significant impact on phenylpropanoid constituents, which emphasizes the need for multi-environment testing to identify seed with phenylpropanoid-dense germplasm for use in plant breeding. Multi-environment testing across diverse agro-ecologies will also reveal which of the environments are more favorable for the production of phenylpropanoid-rich staple grain crops.

## Factors influencing accumulation of phenylpropanoids

Crops can produce a large number of phenolic secondary metabolites that are not essential in the primary process of growth and development, but are of vital significance for their interactions with the environment and for their defense mechanisms (Cheynier et al., 2013). Flavonoids have relevant roles during the establishment of plants in the growing environment (Agati et al., 2013). The production of flavonoids is a response to developmental signals during seed development and to environmental signals, for protection. Thus, flavonoids are involved in protecting crops against major biotic and abiotic stresses (Liu et al., 2013). For some specific flavonoids, there is good understanding of the signals and activation of the phenolic biosynthetic genes (Cheynier et al., 2013). There are various biotic and abiotic stresses that influence the accumulation of specific flavonoids in crops (Supplementary Table 1).

### Abiotic factors

#### Legumes

Drought stress decreased polyphenols in common bean seeds (Ovando-Martínez et al., 2014). Carbon dioxide and water stress increased the isoflavone content of soybean seed, but elevated temperature decreased total isoflavone content by about 65% (Caldwell et al., 2005). Water stress, elevated temperature, and solar radiation led to significant reductions in the specific and total content of isoflavones in soybean (Carrera and Dardanelli, 2015). Likewise, soil drought reduced the total phenolic content in the seeds of pea (*Pisum sativum* L.) and yellow lupin (*Lupinus luteus* L.; Juzoń et al., 2013). In mung bean [*Vigna radiata* (L.) R. Wilczek], water deficit reduced the total phenolics content (Afzal et al., 2014), while elevated UV-B radiation significantly reduced the concentrations of isoflavones and phenolic compounds in soybean seed (Kim et al., 2011). Genotypic differences in response to elevated ozone were noted across mung-bean cultivars, i.e., some cultivars were more sensitive to ozone (O_3_) stress (as measured by differences in antioxidants, metabolites, growth, total biomass, and yield) than others, suggesting the possibility of selection of suitable O_3_ resistant cultivars with improved phenylpropanoids in seeds for areas experiencing high concentation of O_3_ (Chaudhary and Agrawal, 2015).

#### Cereals

In maize, grain flavonoid increased considerably due to water stress, but the accumulation of phenolic compounds and carotenoids decreased (Ali et al., 2010). A marked increase in the total phenolics accumulation was observed in response to drought and salinity, and to a combination of these factors in barley kernels (Ahmed et al., 2013a). Salt increased the nutraceutical quality of mature grains in rice, as measured by total phenolics content, and anthocyanins and proanthocyanins (Chunthaburee et al., 2015), whereas drought led to increased total phenolic acids and carotenoids in wheat grain (Chakraborty and Pradhan, 2012). Stress as a result of nitrogen fertilization increased total free phenolic acids, but decreased conjugated soluble phenolic acids in wheat grain (Stumpf et al., 2015). Rice exposure to high CO_2_ resulted in decreased seed total phenolics content, with the highest reduction in sinapic and *p*-hydroxybenzoic acids. The total flavonoids content also decreased, with apigenin highly affected (Goufo et al., 2014). In whole rice kernels, γ-irradiation led to the accumulation of the main phenolic compounds (e.g., *p*-coumaric acid, ferulic acid), but it decreased anthocyanins (e.g., cyanidin-3-glucoside, peonidin-3-glucoside; Zhu et al., 2010). Wheat exposed to higher levels of solar UV radiation resulted in the production of red kernels and increased the concentrations of phenolic acids, flavonoids, and lutein (Lukow et al., 2012).

### Biotic factors

#### Legumes

Seed flavonoids contribute to a constitutive defense mechanism, and they might accumulate after recurrent infection and as a result of several types of stress (Treutte, 2006). Seed concentrations of flavonoids, alkaloids, and terpenoids define the levels of effectiveness in the control of pathogens and insect pests in most legumes, and especially in common beans (Ndakidemi and Dakora, 2003). Seed accumulation of high amounts of phenolic compounds is toxic to bruchid [*Callosobruchus maculatus* (Fabricius 1775)] and provides resistance to storage pests in cowpea [*Vigna unguiculata* (L.) Walp], chickpea (*Cicer arietinum* L.), and soybean (Sharma and Thakur, 2014). Furthermore, flavonoids and isoflavonoids are considered to have major roles in host plant defense in the Fabaceae family (Mapope and Dakora, 2013). For example, the specific isoflavone content in legumes is strongly related to resistance to pathogens (Treutte, 2006). Rubiales et al. (2015) suggested a prominent role for flavonoid-related compounds in the specific defense against fungi, bacteria, and insects.

#### Cereals

Cell-wall phenolic acids in cereal grains are known to be associated with innate grain resistance to pests and pathogens (Santiago et al., 2013). For instance, the phenolic acids that are accumulated during wheat-kernel development contributed positively to *Fusarium* resistance (McKeehen et al., 1999). Analogous effects of *Fusarium* infection in barley showed that inoculation significantly reduced the ferulic acid content and increased the catechin content in the grain (Eggert et al., 2010). Increased accumulation of phenolic acids in maize pericarp is also associated with weevil [*Sitophilus zeamais* (Motschulsky, 1855)] resistance in tropical genotypes (García-Lara and Bergvinson, 2014). Similar findings in maize were reported for the effects of phenolic compounds against Angoumois grain moths [*Sitotroga cerealella* (Olivier, 1789); Ahmed et al., 2013b]. In contrast, phenolic acids, chlorogenic acids, and tannins were not involved in the infestation and damage caused by rice weevils [*Sitophilus oryzae* (Linnaeus, 1763); Bamisile et al., 2014].

## Phenylpropanoid accumulation in seed

Cereal bran is rich in polyphenols, but these are usually removed from the grain before it is consumed as food. Most of the phytochemicals are lost following milling; thus, there is a trend to increase whole-grain consumption (Schaffer-Lequart et al., 2015). Wheat grain bran and germ contain up to 83% total phenolics, which is 15–18-fold higher on a μmol of gallic acid equiv 100 g^−1^ basis than in the endosperm fraction. Total phenolics progressively decreased during the progress in de-branning from the aleurone layer to the internal portions of the kernel (Adom et al., 2006).

The concentration, type and distribution of flavonoids differs among rice phenotypes. For example, proanthocyanidins are found in red kernels, anthocyanins in black grain, and phenolics in the non-pigmented counterparts (Abdel-Aal et al., 2006; Finocchiaro et al., 2007), whereas anthocyanins are found in the aleurone layer and the pericarp of purple, blue, and red wheat kernels (Havrlentová et al., 2014). Maize with red/ blue and blue kernels often contains a higher proportion of acylated anthocyanins than maize with red and purple kernels. Magenta-colored anthocyanins are concentrated in both the pericarp and aleurone layers, whereas blue maize grains accumulate pigments only in the aleurone layer (Žilić et al., 2012).

Matrix-assisted laser desorption/ionization coupled to imaging mass spectrometry allows simultaneous investigation of the content and spatial distribution of a wide range of biomolecules. Yoshimura et al. (2012) used this mass spectrometry technique to study the distribution of flavonoids in black-pigmented rice seeds, and they identified seven species of anthocyanin monoglycosides and two species of anthocyanin diglycosides. Anthocyanins composed of a pentose moiety (e.g., cyanidin-3-*O*-pentoside, petunidin-3-*O*-pentoside) were found throughout the pericarp, whereas anthocyanins composed of a hexose moiety (e.g., cyanidin-3-*O*-hexoside, peonidin-3-*O*-hexoside) were found only in the dorsal pericarp. Thus, anthocyanin species composed of different sugar moieties have different localization patterns in the pericarp of black rice. Galland et al. (2014) studied the localization, nature, and relative abundance of flavonoids in mature and germinated non-pigmented rice seeds of “Nipponbare” (a *japonica* cultivar) using a combination of confocal microscopy, mass spectrometry and gene expression analysis. They showed that matured rice seed exclusively accumulates flavones mostly in the embryo and to a lesser extent in the pericarp/testa. They detected 21 different flavones. Schaftoside and its two isomers were the major flavones in the embryo (54% of flavonoid compounds, as rhamnetin equivalents seed^−1^). Tricin and its conjugated derivatives accounted for 24% of the flavonoid signal distribution, making these the second largest contributor to the total flavone content of the embryo. In contrast, the pericarp/testa fraction accumulated exclusively schaftoside and two schaftoside isomers. The embryo has both *O*- and *C*-glycosylated flavones, while the pericarp/testa fraction accumulated only *C*-glycosylated flavones. The embryo flavone content is therefore very high when compared with that of the pericarp/testa in “Nipponbare” seeds.

## Genetics and biosynthesis pathways

### Genetics

#### Flavonoids in legumes

The concentration of isoflavones in soybean is a complex multi-genic trait. There are at least 50 quantitative trait loci (QTL) related to this trait (Meksem et al., 2001; Primomo et al., 2005; Gutierrez-Gonzalez et al., 2009, 2011; Zeng et al., 2009; Meng et al., 2011; Yang et al., 2011). Of these, two QTL with main effects that are located in Gm05 (LGA1) and GM08 (LGA2) consistently affected isoflavone content across environments (Gutierrez-Gonzalez et al., 2011). Isoflavone content in soybean is also affected significantly by additive genetic variance (Bi et al., 2015). Gutierrez-Gonzalez et al. (2010) found 35 main-effect genomic regions and many epistatic interactions that control genistein, daidzein, glycitein, and total isoflavone accumulation in soybean seeds. These findings suggest that a complex network of multiple minor-effect loci interconnected by epistatic interactions control isoflavone accumulation in soybean. The magnitude and significance of the effects of many of the nodes and connections in this network varied, however, according to the environment. This study made it possible to identify putative candidate genes for several main-effect and epistatic QTL and for known QTL (Gutierrez-Gonzalez et al., 2010). Wang et al. (2015) noted 34 QTL, of which 23 were new, for both individual and total seed isoflavone contents in soybean; while 6, 7, 10, and 11 QTL were associated with daidzein, glycitein, genistein and total isoflavone, respectively, in multi-generation soybean recombinant inbred lines (RILs; F_5:6_, F_5:7_, F_5:8_). Several DNA markers linked to QTL were identified across environments, thereby indicating that they can be used in the selection of segregants for higher isoflavone content, and also in map-based gene cloning.

Gutierrez-Gonzalez et al. (2010) showed that many enzymes in the phenylpropanoid pathway underlie QTL and modification of genes encoding for enzymes involved in this pathway might promote the biosynthesis of isoflavone in soybean seeds (Hao et al., 2008). Wang et al. (2014) found 33 expression QTL (eQTL) underlying the transcript abundance for the four gene families (*PAL, CHS, IFS, F3H*) on 15 chromosomes. Furthermore, the eQTL between Satt 278-Sat-134, Sat-134-Sct-010, and Satt 149-Sat-234 underlie the expression of both the *IFS* and *CHS* genes. More importantly, they identified five eQTL intervals that overlapped with phenotype QTL (pQTL), and a total of 11 candidate genes within the overlapped eQTL and pQTL.

#### Flavonoids in cereals

Polyphenol compounds that impart red pigment to wheat grain are synthesized through the flavonoid biosynthetic pathways. Himi and Noda (2005) showed that the expression of *CHS, CHI, F3H*, and *DFR* in the flavonoid pathway is completely suppressed indeveloping white grain, but not in red grain, in wheat. All four genes were highly up-regulated in the grain coat tissue of the red lines, whereas there was no significant expression in the white- colored lines, thus indicating that the *R* gene (Myb-type transcription factor) is involved in the activation of early flavonoid biosynthesis genes in wheat (Himi et al., 2005). Flavanone 3-hydroxylase (F3H) is a key enzyme at a divergence point of the flavonoid pathway that leads to the production of different pigments, proanthocyanidin, and anthocyanin. Himi et al. (2011) isolated *F3H-A1, F3H-B1*, and *F3H-D1* on chromosomes 2A, 2B, and 2D of wheat. These genes were highly expressed in red grain and coleoptiles, and they appeared to be controlled by flavonoid regulators in each tissue. Moreover, the telomeric regions of the long arms of the chromosomes of homoeologous group 2 of wheat showed a syntenic relationship to the telomeric region of the long arm of rice chromosome 4, where the rice *F3H* gene is located. To date, a number of structural [*Pal, Chs, Chi, F3h, F3*′*5*′*h, Dfr* (*TaDfr*), *Ans, Mt* (*Fmt*), and *Rt* (*3Rt*)] and regulatory [Myc (*TaMyc*), Myb10 (*Tamyb 10)*, and *Mpc1*] genes are known to be involved in flavonoid biosynthesis in wheat. In most cases, the information on the number of loci involved, chromosomal/intra-chromosomal localization, and sequences (complete or partial) of the gene copies are known (Khlestkina et al., 2015).

Jin et al. (2009) reported two QTL on chromosome 2, as *qPH-2* for phenolic and *qFL-2-1* for flavonoid content, which are flanked by CT87 and G1234, and which show large additive effects that account for 17 and 13%, respectively, of the phenotypic variation in rice. High narrow-sense heritability was estimated using 84 hybrids from an 11-parent diallel mating design, thus showing the importance of additive genetic variance for total phenols in maize (Mahan et al., 2013). A genome-wide association study that involved a global sorghum diversity panel (*n* = 381) and 404,628 SNP markers (Rhodes et al., 2014) identified novel QTL associated with polyphenols in sorghum. Some of these were co-localized with homolog of flavonoid pathway genes from other plants, including an ortholog of maize *Pr1* and a homolog of *Arabidopsis TT16*. General linear models (GLMs) did not precisely map a loss-of-function allele of the *Tannin 1* gene (*tan 1*), while either a GLM accounting for population structure or a standard linear model considering kinship did identify it (Morris et al., 2013). Furthermore, *tan 1* was accurately mapped using a simple loss-of-function genome scan for the genotype-phenotype co-variation only in the putative loss-of-function allele.

#### Anthocyanins in cereals

The deposition of proanthocyanidins in the seed testa results in red grain whereas anthocyanins in the pericarp and aleurone layer give rise to purple and blue colored wheat kernels, respectively (Zeven, 1991). *Ba1* (Keppenne and Baenziger, 1990) and *Ba2* (Dubcovsky et al., 1996) were found to control blue grain in a tall wheat grass [*Thinopyrum ponticum* (hereonward referred as *Th. ponticum*) = *Agropyron elongatum*] and in *Triticum monococcum*, respectively. These two genes were physically mapped: *Ba1* at FL0.71-0.80 on chromosome 4Ag (Zheng et al., 2006), and *Ba2* near the centromere on chromosome 4AL (Dubcovsky et al., 1996). The wild relative *Th. bessarabicum* bears the gene *BaThb*, which produced blue grain and has been physically mapped between the centromere and FL0.52 on chromosome arm 4JL. *BaThb* differs from *Ba1* and *Ba2*, and has a strong dose effect, thus confirming *Th. bessarabicum* as another source of blue aleurone grain in wheat (Shen et al., 2013). To date, several blue wheat elite lines have been developed. These lines carry *Th. ponticum* or *T. monococcum* introgressed chromosomes. Burešováet et al. (2015) found that 17 of 26 such lines have introgression from *Th. ponticum*, while the remaining bear *T. monococcum* chromatin. This finding suggests that these blue aleurone wheat lines show major differences in chromatin composition. Introgression activates the blue aleurone trait, which is inactivated in bread wheat germplasm lacking the *Th. ponticum* chromosome segment.

The genes *Pp1* and *Pp3* mapped on the short chromosome arms of the homeologous group 7 and on chromosome arm 2AL, respectively, control purple grain color in wheat (Dobrovolskaya et al., 2006; Khlestkina et al., 2010; Tereshchenko et al., 2012). Furthermore, the *Pp1* genes are orthologs to both maize *C1* and rice *OsC1*, and encode MYB-like transcription factors that activate structural genes related to enzymes associated with anthocyanin biosynthesis (Khlestkina, 2013). *Pp3* is orthologous to *Ra* in rice (Wang and Shu, 2007) and *Lc* in maize (Ludwig et al., 1989). It encodes *TaMYC1*, which is strongly expressed in the pericarp (Shoeva et al., 2014). *Pp1* and *Pp3* upregulate the transcript abundance of structural genes *Chi* (Chalcone-flavone isomerase) and *F3h* (flavanone 3-hydroxylase) in the pericarp of near isogenic lines carrying various combinations of *Pp* alleles (Gordeeva et al., 2015).

The pericarp of red rice grains accumulates proanthocyanidin (Sweeney et al., 2006), while purple rice grain accumulate anthocyanin (Rahman et al., 2013). Maeda et al. (2014) confirmed that *Pp* on chromosome 1 and *Pb* on chromosome 4 acted together to influence grain color (Wang and Shu, 2007; Rahman et al., 2013). They also indicated that *Kala1, Kala3*, and *Kala4* are essential for black pigmentation. Their loci were mapped between RM7405 and RM7419 on chromosome 1, between RM15008 and RM 3400 on chromosome 3, and between RM1354 and RM7210 on chromosome 4, respectively. Ectopic expression of the *Kala4 bHLH* gene leads to expression of anthocyanin biosynthesis genes in the pericarp, and produces black rice grains, while a DNA duplication event at the 5′-end of the gene that correlated with *kala4* expression also controls black grain (Oikawa et al., 2015).

Wei et al. (2013) showed that in barley a dominant gene *Blp* mapped on chromosome 1HL controls black grain, while the complementary dominant genes *Pre1* and *Pre2* mapped on chromosome 2HL determine the purple color. They also indicated that the complementary dominant genes *Blx1, Blx3*, and *Blx4* mapped on chromosome 4H, plus *Blx2* and *Blx5* mapped on chromosome 7HL, are responsible for blue colored barley kernels.

Anthocyanin biosynthesis in maize is regulated by interactions between two sets of transcription factors that are encoded by *c1/pl1* and *r1/b1*; *c1* and *r1* regulate pigmentation in the kernel aleurone, and *pl1* and *b1* regulate it in the plant body (Chandler et al., 1989). The *pr1* gene has a role in the *cl*- and *rl*-regulated anthocyanin biosynthesis pathway (Sharma et al., 2011). The *pr1* locus accumulates red (pelargonidin) and the *Pr1* accumulates purple (cyanidin) anthocyanins in the aleurone cells of seeds. The putative F3′H encoding gene (*Zmf3*′*h1*) was mapped on chromosome 5L, while purple and red anthocyanins accumulated in *Pr1* and *pr1* lines, respectively. Furthermore, *pr1* has four alleles, which are characterized by insertion or deletion polymorphisms that co-segregated with the red aleurone phenotype in the F_2_ population containing *Pr1* and *pr1* alleles. This gene is under the regulatory control of anthocyanin transcription factors *red1* and *colorless1*. Moreover, Sharma et al. (2012) showed that *Zmf3*′*h1* also participates in biosynthesis of phlobaphenes and 3-deoxyflavonoid compounds, which accumulate in maize pericarp and cob glumes and silks, and are under regulatory control by *P1*. Thus, *Zmf3*′*h1* has a significant role in generation of diversity for anthocyanin, phlobaphenes, 3-deoxyanthocyanidin and C-glycosyl flavone compounds; the latter two of these compounds impart maize plant resistant to pests and pathogens (Nicholson and Hammerschmidt, 1992; Byrne et al., 1996).

*PERICARP COLOR 1* (*P1*) is an R2R3-MYB type transcription factor that controls the accumulation of brick red phlobaphenes pigments in grain pericarp in maize. Phlobaphenes are polymers of the flavan-4-ols apiforol and luteoforol, and are generated from naringenin or eriodictyol by dehydroflavonol reductase (DFR), which is encoded by maize *A1*. *P1* alleles specify different pericarp and cob glume colors. For example, *P1-ww* results in white pericarps and white cob glumes, whereas *P1-rr* produces red pericarps and red cob glumes. *A1* mutants in a *P1-rr* background (*P1-rr; a1*) display an unidentified brown pigment that contrasts with the white *P1-ww* pericarp, thereby suggesting metabolic shunting toward a different branch of the flavonoid pathway (Casas et al., 2014). Most of the elite lines used in the production of hybrid maize lack flavones. Casas et al. (2014) showed that maize lines harboring the *P1-rr* allele in combination with recessive *a1* accumulate flavones to the same levels as flavone-rich vegetables. These results suggest that nutritionally beneficial flavones can be re-introduced into elite lines to increase the dietary benefits of maize.

Clearly, over the years a greater understanding of phenylpropanoid genetics has been achieved, and this knowledge-based inheritance can now facilitate the enriching of staple grain crops with health-promoting compounds.

### Biosynthesis pathways

**Figure 1:**
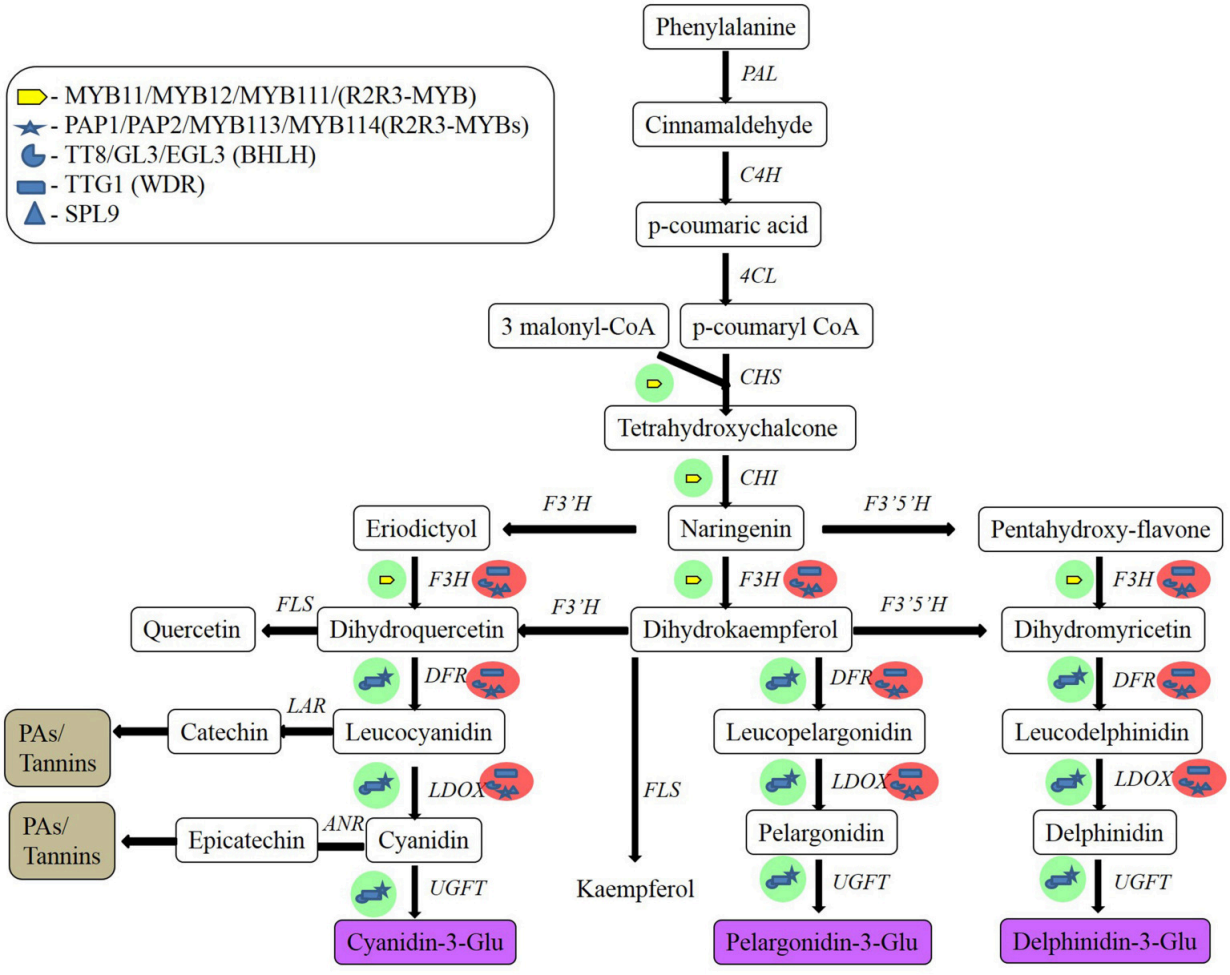
**Flavonoid biosynthetic pathway in plant cells and regulatory gene regulation of this pathway in ***Arabidopsis*****. Green and red circles indicate activation and repression, respectively. MYB, myeloblast; PAP, production of anthocyanin pigment; TT8, transparent testa A8; bHLHs, basic helix-loop-helix proteins; TTG1, transparent testa glabrous1; SPL9, squamosa promoter binding protein-like 9; PAL, phenyalanine ammonia-lyase; C4H, cinnamic acid 4-hydroxylase; 4CL, 4-coumarate CoA ligase; CHS, chalcone synthase; CHI, chalcone isomerase; F3H, flavanone 3′-hydroxylase; F3′H, flavonoid 3′-hydroxylase; F3′5′H, flavonoid 3′5′-hydroxylase; DFR, dihydroflavonol reductase; LDOX, leucoanthocyanidin oxidase; UGFT, UDP-glucose flavonoid 3-O-glucosyl transferase; FLS, flavonol synthase; LAR, leucoanthocyanidin reductase; ANR, anthocyanidin reductase (After Pandey et al., 2014).

Flavonoid biosynthesis (Figure 1) begins with the phenylpropanoid pathway, in which phenylalanine is converted into p-coumaroyl CoA. This pathway is mediated by the flavonoid metabolon, which is attached to the cytoplasmic face of the endoplasmic reticulum. Metabolons are multienzyme complexes. They represent highly organized assemblies of sequential enzymes in a metabolic pathway, and they provide increased metabolic efficiency and higher substrate selectivity (Kaur-Sawhney et al., 2003). The basic carbon structure of flavonoids is generated by a two-step condensation process that is mediated by chalcone synthase (CHS) and chalcone isomerase (CHI). The resulting colorless naringenin is then oxidized by F3H to dihydrokaempferol. Naringenin can also be directly hydroxylated to yield dihydroflavonols, and then later converted into anthocyanidins. Despite the central biosynthetic pathway being conserved in plants, various enzymes can modify the basic flavonoid skeletal structure including reductases, isomerases, hydroxylases, and dioxygenases, to form different subclasses of flavonoids in different species. Transferases add groups like sugars, methyl, or acyl groups to the backbone structure. The synthesis of proanthocyanidins branches off from the anthocyanin pathway subsequent to the reduction of dihydroquercetin to leucocyanidin. The two major enzymes involved in the formation of proanthocyanidins are leucoanthocyanidin reductase (LAR) and anthocyanidin reductase (ANR) (Bogs et al., 2005).

The flavonoid biosynthesis pathway is extensively regulated by transcription factors, such as the MYB proteins, basic helix-loop-helix (bHLH) factors, and WD-repeat-containing proteins. Transcriptional regulation has been extensively investigated in maize and *Arabidopsis*. This has facilitated the identification of differences in regulation between monocots and dicots (Ferreyra et al., 2012). The MYB domain is made up of one (MYBR1), two (R2R3-MYB), or three (MYB3) repeats of about 52 amino acids, with R2R3-MYB being the most predominant. Specific motifs and conserved residues mean that these proteins can regulate single branches of the flavonoid pathway (Hichri et al., 2010; Lin-Wang et al., 2010). The overexpression of VlMYBA-1 in the hair roots of grapevine induces the expression of only the genes involved in anthocyanin biosynthesis and transport, whereas the overexpression of VvMYBPA1 and VvMYBPA2 selectively activates genes involved in the synthesis of proanthocyanidins. Albert (2015) isolated *Tr-MYB133* and *Tr-MYB134* in white clover (*Trifolium repens* L.), which encode R2R3-MYBs that antagonize the activity of MBW activation complexes. These two genes are also conserved in other legume species, and form two subclades within the larger anthocyanin/proanthocyanidin clade of MYB repressors. However, unlike petunia (*Petunia* sps.) and *Arabidopsis*, these R2RS-MYB repressors do not prevent ectopic accumulation of anthocyanins or proanthocyanidins. Instead, they are expressed when anthocyanins or proanthocyanidins are synthesized, and provide feedback regulation to MBW complexes. This feedback occurs because *Tr-MYB133* and *Tr-MYB134* are themselves regulated by MBW complexes. *Tr-MYB133* is regulated by MBW complexes that contain anthocyanin-related R2R3-MYB proteins (Tr-RED LEAF), while *Tr-MYB134* is regulated by complexes containing the proanthocyanidin R2RS-MYBs (Tr-MYB14). Thus, regulation of *Tr-MYB133* and *Tr-MYB134* by pathway-specific MBW complexes results in anthocyanin or proanthocyanidin synthesis (Albert, 2015).

The bHLH proteins are ubiquitous transcription factors that are found across eukaryotes, from yeast to human. They are widely distributed in plants and are characterized by the presence of a critical region, called the bHLH domain. The basic region of the bHLH domain consists of 15–17 amino acids, and this is responsible for DNA binding and activation. The bHLH proteins form heterodimers and function as repressors in the absence of the basic region (Toledo-Ortiz et al., 2003). The first 200 amino acids of the protein on the N'-terminal are referred to as MIR (MYB–interacting region), and the next 200 amino acids are known as the WD-40/AD domain, which facilitates the formation of ternary complexes known as MBW complexes in plant species. The bHLH proteins can bind DNA as a single molecule or as a dimer, with MYB proteins based on the promoter target (Hichri et al., 2011). Unlike MYB proteins, some bHLH proteins can influence more than one branch of the flavonoid pathway. The TT8 (transparent testa 8) factor of *Arabidopsis* is an example of the regulation of both the anthocyanin and proanthocyanidins pathways.

Ternary complexes that comprise the above-mentioned transcription factors that are referred to as MBW complexes have been comprehensively identified in model plants and crops (Supplementary Table 2). In *Arabidopsis*, the MYB protein controls the target-gene specificity of the ternary complex. The presence of PAP1/PAP2 (production of anthocyanin pigment), TT2 (transparent testa 2), GL1 (glabrous 1), WER (werewolf), and AtMYB61 regulate anthocyanin accumulation in seedlings, proanthocyanidins biosynthesis in seed integuments, trichome formation, root-hair initiation, and mucilage production in seed integuments, respectively (Baudry et al., 2004). Flavonoid biosynthesis is also regulated by environmental factors, such as light and external stresses in grape (*Vitis vinifera* L.; Li et al., 2014), and the intensity of light and sucrose conditions in *Arabidopsis* (Das et al., 2012), which is mediated by the MYBL2 factor. In mulberry (*Morus* spp.), transcriptional levels of regulatory genes involved in the biosynthesis of anthocyanins are directly related to the degree of ripening and the coloration intensity of the fruits (Qi et al., 2014).

In summary here, elucidating the intricate regulatory patterns of the flavonoid biosynthesis pathway will pave the way for genetic enhancement. Understanding how biosynthetic enzymes are regulated and their spatio-temporal organization will enable modification of the patterns of flavonoid expression and accumulation. Manipulation of the pathways can generate fruit and vegetables enriched in antioxidant and nutritional compounds, as well as provide other medicinal benefits. Future research should be aimed at delineation of the factors that control the expression of the regulatory genes, and also at understanding the allelic variability between cultivars of the same species to identify useful DNA markers as aids for indirect selection in breeding.

## Germplasm mining for variations in phenylpropanoids

### Flavonoids in legumes

A systematic search reveals that there have been limited germplasm accessions evaluated for flavonoids among grain legume crops (Table 1). Up to two-fold variations in flavonoid content were noted in cowpea, groundnut (*Arachis hypogaea* L.), guar [*Cyamopsis tetragonoloba* (L.) Taub.], mung bean, and soybean germplasm. The differences in chickpea and lima bean (*Phaseolus lunatus* L.) germplasm were 76- and 86-fold, respectively. In common bean, 2–13-fold differences were reported among landraces and wild and weedy types, with the latter showing maximum fold differences in flavonoid content. Kaempferol and quercetin were the main flavonoid compounds in common bean (Espinosa-Alonso et al., 2006; Mishra et al., 2012). Four-fold differences were found in cowpea and 6–76-fold in common bean germplasm. In cowpea, seed color and content of flavonol were correlated, with red-seeded accessions containing greater flavonol than white-seeded cowpeas. Other seed color types had limited variations, except for light-brown-seeded accession IAR 48, which showed exceptionally high flavonol content [0.796 mg g^−1^ dry weight (DW)] among light-brown-seeded cowpeas (Ojwang et al., 2012). Quercetin was the most abundant flavonol in cowpea (Ojwang et al., 2012), while kaempferol and quercetin flavonol were the most abundant flavonols in common bean (Doria et al., 2012).

**Table 1:** **Germplasm-wide variations in total phenylpropanoid constituents in food legumes**.

**Germplasm (no.)**	**Variation for flavonoids**	**References**
**FLAVONOID**		
**Common bean (*****Phaseolus vulgaris*** **L.)**		
Landraces (20)	0.05–0.41 mg quercetin (QUE) equivalent (QUAE) g^−1^ DW	Mishra et al., 2012
Wild and weedy types (64)	0.008–0.106 mg g^−1^ FW, G 12896-B and G 11025B being highest	Espinosa-Alonso et al., 2006
Zolfino landraces (4)	0.302–0.711 mg g^−1^ FW	Romani et al., 2004
**Chickpea (*****Cicer arietinum*** **L.)**		
Landraces (20)	0.05–0.41 mg quercetin equivalent (QUAE) g^−1^ DW	Mishra et al., 2012
**Cowpea (*****Vigna unguiculata*** **(L.) Walp.)**		
Black, red, tan, and white grains (8)	0.253–0.442 mg g^−1^ DW, quercetin being highest (0.214–0.279 mg)	Wang et al., 2008
**Groundnut (*****Arachis hypogaea*** **L.)**		
Black, pink, red, tan, and white grains (8)	0.138–0.336 μg g^−1^ DW, quercetin being highest (0.133–0.288 mg)	Wang et al., 2008
**Guar (*****Cyamopsis tetragonoloba*** **(L.) Taub.)**		
Accession (36)	13–23 mg 100 g^−1^ DW; Kaempferol, the major component (10.7–19.8 mg)	Wang and Morris, 2007
**Lima bean (*****Phaseolus lunatus*** **L.)**		
Black, brown, pink, red, and white-grains (50)	0.2–17.3 mg rutin equiv. (RUE) g^−1^ DW	Agostini-Costa et al., 2015
**Mung bean (*****Vigna radiata*** **(L.) R. Wilczek)**		
Accession (50)	1.204–2.932 mg g^−1^ DW	Kim et al., 2013
**Soybean (*****Glycine max*** **(L.) Merr.)**		
Black, brown, green, red, and yellow grains (8)	0.892–0.916 mg g^−1^ DW; genistein (0.438–0.458 mg) and daidzein (0.315–0.354 mg) greater than Kaempferol (0.038–0.068 mg)	Wang et al., 2008
**FLAVONOL**		
**Common bean**		
Gene pools differing in seed color (16)	0.002–0.125 mg g^−1^ DW	de Lima et al., 2014
Landrace-based populations (10)	0.011–0.081 mg g^−1^ DW	Doria et al., 2012
Zolfino landraces (4)	1.18–7.09 mg g^−1^ FW	Romani et al., 2004
**Cowpea (*****Vigna unguiculata*** **(L.) Walp.)**		
Black, brown, green, golden, and white grains (10)	0.27–1.06 mg g^−1^ DW, red-seeded had greater (mean 0.97 mg) than white-seeded (0.27 mg)	Ojwang et al., 2012
**ISOFLAVONE**		
**Common bean**		
Gene pools differing in seed color (16)	0.0008–0.14 mg g^−1^ DW	de Lima et al., 2014
Landrace-based populations (10)	0.009–0.113 mg g^−1^ DW	Doria et al., 2012
Zolfino landraces (4)	0.002–0.015 mg g^−1^ FW	Romani et al., 2004
**Soybean**		
Indian and exotic accessions (46)	0.234–2.092 mg g^−1^ DW	Kumar et al., 2015
0 to VI maturity groups (40)	0.551–7.584 mg g^−1^ DW	Zhang et al., 2014
Cultivars (44)	0.276–1.709 mg g^−1^ DW	Kim et al., 2014
Seed size variations (204)	0.682–4.778 mg g^−1^ DW	Kim et al., 2012b
0 to II maturity groups (210)	1.161–2.743 mg g^−1^ DW	Wang et al., 2000

*Original data on phenylpropanoid constituents given in papers cited here were converted and presented into mg g^-1^ dry weight*.

Multi-fold differences were noted in isoflavone content among common bean germplasm, with black-, ivory-, and brown-yellow-seeded accessions showing the highest isoflavones (Doria et al., 2012; de Lima et al., 2014). There were 2–14-fold differences in isoflavone content in soybean. Daizin, genistin, glycitin, malonyldaidzin, malonylglycitin, and malonylgenistin were the major moieties (Kim et al., 2012a; Zhang et al., 2014). Maturity groups (MG) had significant differences in isoflavone content in soybean. For example, MG V and VI had significantly higher total isoflavones compared to MG 0 to IV. Differences among MG 0 to IV or between MG V and VI were, however, not statistically significant (Zhang et al., 2014). Seed size influenced mean isoflavone content among geographically different soybean accessions. For example, small-seeded accessions from North America and Korea had similar isoflavone (2.53, 2.56 mg g^−1^, respectively), the medium-seeded accessions from North America and Korea had higher isoflavone (2.24, 2.48 mg g^−1^, respectively) than those from China (1.38 mg g^−1^), while large-seeded Korean accessions had higher isoflavone (1.83 mg g^−1^) levels than those from North America and China accessions (1.20, 1.34 mg g^−1^, respectively; Kim et al., 2012b).

### Flavonoids in cereals

Barley, rice, sorghum, and wheat germplasm/cultivars have been studied for variations in flavonoids (Table 2). About 2-fold variation among barley and up to 21-fold variation among rice germplasm were noted. Black-grained and red-grained rice accessions had greater flavonoids than white-grained types (Shen et al., 2009; Shao et al., 2014a). Wheat germplasm that differed in grain color had relatively narrow genetic differences for flavonoids. Up to 12-fold difference in flavone and up to 6-fold difference in flavanone content were observed among sorghum open-pollinated and hybrid cultivars.

**Table 2:** **Germplasm-wide variations in total phenylpropanoid constituents in staple cereals**.

**Germplasm (no.)**	**Variation for flavonoids**	**References**
**FLAVONOID**		
**Barley (*****Hordeum vulgare*** **L.)**		
Landraces (37)	0.47–1.23 mg catechin equival. (CE) mg g^−1^ dry weight (DW)	Abidi et al., 2015
Hulled and hull-less (11)	27–66 mg quercetin equiv. (QUE) g^−1^ extract	Mahmoudi et al., 2015
**Rice (*****Oryza sativa*** **L.)**		
Diverse accessions (20)	0.19–3.28 and 0.20–3.54 mg CE g^−1^ DW in two seasons	Shao et al., 2014a
Cultivars with pigmented and non-pigmented grains (11)	0.0012–0.0258 mg QE g^−1^ bran; higher flavonoid in pigmented than non-pigmented; greater flavanol in black-colored *indica* than black-colored *japonica*	Huang and Ng, 2012
Black, red, and white grains (481)	0.89–2.86 mg Rutin equiv. (RE) g^−1^ DW, average values greater in black (0.24 mg) than red (0.15 mg) and white (0.13 mg) grains	Shen et al., 2009
**Wheat (*****Triticum aestivum*** **L.)**		
Black, purple, and white grains (4)	0.236–0.319 mg RE g^−1^ DW, with black grains being highest in flavonoid	Li et al., 2015
**FLAVONE AND FLAVANONE**		
**Sorghum (*****Sorghum bicolor*** **(L.) Conrad Moench)**		
Colored grains (12)	Flavone: 0.008–0.1 mg g^−1^ DW; flavanone: 0.008–0.048 mg g^−1^ DW	Dykes et al., 2009
Black grained lines and hybrids (8)	Flavone: 0.018–0.056 mg g^−1^ DW; flavanone: 0.089–0.119 mg g^−1^ DW	Dykes et al., 2013

*Original data on phenylpropanoid constituents given in papers cited here were converted and presented into mg g^-1^ dry weight*.

### Anthocyanin in legumes

Nine-fold differences in anthocyanin levels were noted among common bean germplasm lines differing in seed color and weight. Accessions with brown, red, or black seed color had higher levels of anthocyanins (0.003–0.005 mg g^−1^ DW; Akond et al., 2011). Kidney-bean germplasm showed large range variations in total anthocyanins (0.07–2.78 mg g^−1^ DW), black-seeded types being richer sources of anthocyanins than red- or brown-seeded types (Choung et al., 2003). About 2-fold differences in total anthocyanin were recorded among cowpea lines. Black-seeded cultivars had higher levels of anthocyanins than the green-seeded type. The predominant anthocyanin compounds include delphidin-3-*O*-glucoside, cyanidin-*O*-glucoside, petunidin-*O*-glucoside, and malvidin-*O*-glucoside (Ojwang et al., 2012). In soybean, several-fold differences (30–213 times) were noted among Chinese and Japanese cultivars and landraces, with cyanidin-3-glucoside being the most abundant (Zhang et al., 2011; Phommalath et al., 2014).

### Anthocyanins in cereals

Variations in anthocyanin levels in germplasm/cultivars have been reported for barley, maize, rice, sorghum, and wheat (Table 3). Two-fold to eighty-fold variation in anthocyanin concentration was noted among barley germplasm. Purple-grain and blue-grain barley groups had significantly greater mean anthocyanin levels (0.32 mg g^−1^) than black barley (0.04 mg g^−1^). The most common anthocyanins in purple barley were cyanidin-3-glucoside, peonidin-3-glucoside, and pelargonidin-3-glucoside, whereas delphinidin-3-glucoside was the most abundant anthocyanin in blue and black barley groups (Kim et al., 2007). The predominant anthocyanins were delphidin-3-malonylglucoside and cyanidin-3-malonylglucoside, followed by delphidin-3-glucoside and cyanidin-3-glucoside in blue barley (Diczházi and Kursinszki, 2014). In maize, multi-fold differences in total anthocyanins, with most reporting 15–36-fold were observed among colored-grain accessions. Cyanidin-glucoside was the major anthocyanin (Lopez-Martinez et al., 2009; Kuhnen et al., 2011; Mendoza-Díaz et al., 2012; Žilić et al., 2012). Maize with dark-red, blue, or purple grain colors holds immense promise for the development of functional foods and natural colorants. Several-fold differences, which ranged from 4 to 121 times, were noted among pigmented rice germplasm. Cyanidin-3-glucoside, peonidin-3-glucoside, cyanidin diglucoside, and malvidin were the major anthocyanins in black-rice and red-rice kernels (Ryu et al., 1998; Abdel-Aal et al., 2006; Lee, 2010; Zhang et al., 2010; Chen et al., 2012). Red-grained and black-grained sorghum cultivars showed up to 21-fold differences in anthocyanins.

**Table 3:** **Germplasm-wide variations in total phenylpropanoid constituents in staple cereals**.

**Germplasm (no.)**	**Variation in total anthocyanin content**	**References**
**Barley (*****Hordeum vulgare*** **L.)**		
Colored grains (4)	0.047–0.084 mg g^−1^ dry weight (DW)^$^	Diczházi and Kursinszki, 2014
Hulled and unhulled colored grains (127)	0.013–1.038 mg catechin equiv. g^−1^ DW	Kim et al., 2007
**Maize (*****Zea mays*** **L.)**		
Blue-grain hybrids/varieties (7)	0.65–1.05 mg cyaniding 3-glucoside (Cy3Glu) equiv. g^−1^ DW	Urias-Lugo et al., 2015
Waxy maize (49)	0.07–1.06 mg Cy3Glu equiv. g^−1^ DW	Harakotr et al., 2015
Colored grains (4)	1.74–9.63 mg Cy3Glu equiv. g^−1^ DW	Mendoza-Díaz et al., 2012
Colored grains (10)	0.002–0.696 mg Cy3Glu equiv. g^−1^ DW	Žilić et al., 2012
Red and blue grains (9)	0.02–0.72 mg Cy3Glu equiv. g^−1^ DW	Montilla et al., 2011
Waxy colored and normal yellow grains (3)	0.001–2.761 mg Cy3Glu equiv. g^−1^ DW	Hu and Xu, 2011
Colored grains (18)	0.30–8.50 mg Cy3Glu equiv. g^−1^; purple, 0.93 to8.50 mg; black, 0.76-1.20 mg; Red, 0.85–1.54 mg	Lopez-Martinez et al., 2009
Colored grains (9)	0.051–1.277 mg g^−1^ DW^$^	Abdel-Aal et al., 2006
**Rice (*****Oryza sativa*** **L.)**		
Colored grains (9)	0.21–2.98 mg g^−1^ DW^$^	Chen et al., 2012
Black and red grains (13)	Black grains: 1.09–2.56 mg Cy3Glu equiv. 100 g^−1^ DW; Red grains: 0.003–0.014 mg Cy3Glu equiv. g^−1^ DW	Sompong et al., 2011
Black grains (12)	12.31–51.01 mg Cy3Glu equiv. g^−1^ DW	Zhang et al., 2010
Black grains (10)	0.052–1.684 mg Cy3Glu equiv. g^−1^ DW	Lee, 2010
Wild rice with colored grains (3)	0.027–3.276 mg g^−1^ DW^$^	Abdel-Aal et al., 2006
**Sorghum (*****Sorghum bicolor*** **(L.) Conrad Moench)**		
Colored grains (12)	0.032–0.68 mg g^−1^ DW^$^	Dykes et al., 2009
Black grained lines and hybrids (8)	0.33–1.05 mg g^−1^ DW^$^	Dykes et al., 2013
**Wheat (*****Triticum*** **species)**		
Durum and bread wheat colored grains (76)	Blue colored bread wheat, 0.082–0.174 mg g^−1^ DW, mean 0.118 mg; purple and red colored durum wheat, 0.008–0.05 (mean, 0.023), and 0.001–0.025 mg (mean, 0.01), respectively^$^	Ficco et al., 2014
Colored grains (4)	0.007–0.12 mg g^−1^ DW^$^	Žofajova et al., 2012
Pigmented grains (13)	0.0034–0.0752 cyanidin glucoside equiv. mg g^−1^ DW	Eticha et al., 2011
Colored grains (7)	0.007–0.212 mg g^−1^ DW^$^	Abdel-Aal et al., 2006

*Original data on phenylpropanoid constituents given in papers cited here were converted and presented into mg g^-1^ dry weight*.

$ *total anthocyanin (not the anthocyanin compounds) value was given in the original literature*.

Wheat germplasm lines and cultivars showed up to 30-fold difference in anthocyanin content. Blue and purple grain accessions had higher levels of anthocyanins (Abdel-Aal et al., 2006; Eticha et al., 2011; Žofajova et al., 2012; Ficco et al., 2014). Five to eight anthocyanin compounds were noticed in blue grain wheat extracts, compared to three anthocyanin compounds in purple and red wheat (Ficco et al., 2014). Delphinidin-3-*O*-rutinoside, delphinidin 3-*O*-glucoside, and malvidin-3-*O*-glucoside were the predominant anthocyanins in blue wheat, while cyanidin-3-*O*-glucoside, peonidin-3-*O*-glucoside, and malvidin-3-*O*-glucoside were found in purple wheat (Abdel-Aal et al., 2006; Ficco et al., 2014). Zeven (1991) gave details on the origin and history of wheat with blue and purple grains.

### Phenolics in legumes

Table 4 gives the variations reported for phenolics in grain legumes. Among lima bean germplasm, there was 2–4-fold variation for phenols, except for one study that indicated a very high level (97-fold; Agostini-Costa et al., 2015). Ferulic acid was the most abundant, followed by p-coumaric and sinapic acids (Espinosa-Alonso et al., 2006; Luthria and Pastor-Corrales, 2006). Black, brown and red common beans had higher phenols than white grain types (Akond et al., 2011; Agostini-Costa et al., 2015). Up to 13-fold differences in total phenols were noted among chickpea germplasm with colored grains. *Desi* types had higher levels of phenols compared to *Kabuli* types. Among the anatomical parts, the seed coat was the major source of variation for total phenols. Cowpea germplasm and cultivars showed 3–11-fold differences in total phenols. Protocatechuic acid was the major phenolic, while p-hydroxybenzoic, caffeic, p-coumaric, ferulic, 2,4-dimethoxybenzoic, and cinnamic acids were also reported (Cai et al., 2003). Valencia groundnut (var. *fastiggiata*) differing in seed color showed 34-fold differences in total phenols. Seeds with pink color had significantly higher levels of phenols than those with gray and yellow color. The major phenolic compounds in the testae of nearly all genotypes were *p*-coumaric and vanillic acids. Korean mung bean germplasm had 5-fold differences in total phenols. Kim et al. (2013) detected a total of 25 phenolic compounds in mung bean germplasm, with rutin being predominant, and its concentration among these germplasm varied from 1.09 to 2.72 mg g^−1^.

**Table 4:** **Germplasm-wide variations in phenylpropanoid constituents in food legumes**.

**Germplasm (no.)**	**Variation in total phenols**	**References**
**Common bean (*****Phaseolus vulgaris*****)**		
Black, brown, pink, red, and white-grains (50)	0.1–9.7 mg GAE g^−1^ DW	Agostini-Costa et al., 2015
Landrace-based populations (10)	0.007–0.032 mg GAE g^−1^ DW	Doria et al., 2012
Varying in seed color and weight (29)	6–14 mg g^−1^ GAE DW	Akond et al., 2011
Wild and weedy types (64)	50–131 mg kg^−1^ GAE fresh weight	Espinosa-Alonso et al., 2006
Market types (15)	0.19–0.48 mg g^−1^ GAE DW	Luthria and Pastor-Corrales, 2006
**Chickpea (*****Cicer arietinum*** **L.)**		
Colored grains (17)	0.2–32.6 mg catechin equiv. (CAE) g^−1^ DW; seed coat the major source of phenolics	Segev et al., 2010
**Cowpea (*****Vigna unguiculata*** **(L.) Walp.)**		
Brown and white-grained (7)	0.85–2.95 mg GAE g^−1^ DW	Noubissié et al., 2012
Cultivars (17)	0.35–3.77 mg g^−1^ DW	Cai et al., 2003
**Groundnut (*****Arachis hypogaea*** **L.)**		
Gray, pink, purple, red, yellow, and variegated colored Valencia's (15)	Seed testa: 2.5–84.5 mg GAE g^−1^ DW; significantly greater phenols among accessions with pink grain color	Khaopha et al., 2012
Mung bean (*Vigna radiata* (L.) R. Wilczek)		
Germplasm (56)	0.12–0.59 mg g^−1^ DW	Kim et al., 2013
**Soybean (*****Gylcine max*** **(L.) Merr)**		
Black grains Japanese cultivars and landraces (227)	75–380 and 19–389 mg GAE g^−1^ DW in two seasons; more phenols in purple flowers than white flowers producing cultivars	Phommalath et al., 2014
Seed size variation (204)	0.65–5.22 mg g^−1^ DW	Kim et al., 2012b
Black grains (60)	5.12–60.58 mg GAE g^−1^ DW	Zhang et al., 2011

*Original data on phenylpropanoid constituents given in papers cited here were converted and presented into mg g^-1^ dry weight*.

Soybean germplasm from landraces and cultivars from China, Korea, and the USA showed 8–12-fold variations in total phenols. US soybean germplasm had higher mean total seed phenols (2.73 mg GAE g^−1^) than those from Korea (1.98 mg g^−1^) and China (1.680 mg g^−1^) (Kim et al., 2012b), as for black-grain Chinese germplasm (23.57 mg g^−1^; Zhang et al., 2011). Furthermore, the total phenols varied from 0.93 to 5.56 mg g^−1^ in North American soybean, 0.72 to 4.21 mg g^−1^ in Chinese soybean, 0.65 to 5.07 mg g^−1^ in Korean soybean (Kim et al., 2012b), 53 to 384 mg GAE g^−1^ in Japanese soybean (Phommalath et al., 2014) and 5.12 to 6.06 mg g^−1^ in black-grain Chinese germplasm (Zhang et al., 2011). Grain-size variations had significant effects on phenols, with higher mean total phenols in small- (2.24 mg g^−1^) as compared to medium- (1.93 mg g^−1^) and large- (1.95 mg g^−1^) grain types (Kim et al., 2012b). This suggests that phenolic compounds are condensed in small-grain soybean, whereas they appear diffused at lower densities in large-grain soybean (Kim et al., 2012b).

### Phenolics in cereals

Cereal germplasm and cultivars have been the most extensively studied for variations in phenolics (Table 5). Two-fold to three-fold variations were noted for total phenols in barley. Catechine, *p*-coumaric, and ferulic acids were the most abundant (Dvořáková et al., 2008; Abdel-Aal et al., 2012; Gamel and Abdel-Aal, 2012). Genotypic differences for specific groups were also observed. For example, protocatechuic and caffeic acids were found in Egyptian hulled cultivars, but not in Canadian hulled cultivars (Gamel and Abdel-Aal, 2012). Blue-grain accessions had higher mean phenolics (7.73 mg g^−1^ DW) than white (6.69 mg g^−1^), purple (6.14 mg g^−1^), and black (5.61 mg g^−1^)-grain barley types (Siebenhandl-Ehn et al., 2011). Kim et al. (2007) also reported higher mean phenolics (0.27 mg g^−1^ DW) in blue and purple barley than in black-grained barley (0.21 mg g^−1^). Furthermore, a large range for total phenolics was noted within each group, which suggested that there are accessions with high phenolics in each color group (Siebenhandl-Ehn et al., 2011; Abdel-Aal et al., 2012).

**Table 5:** **Germplasm-wide variations in total phenylpropanoid constituents in staple cereals**.

**Germplasm (no.)**	**Variation in total phenols**	**References**
**Barley (*****Hordeum vulgare*** **L.)**		
Landraces (37)	0.70–1.95 mg gallic acid equivalents (GAE) g^−1^ dry weight (DW)	Abidi et al., 2015
Hulled and hull-less (11)	0.06–0.14 mg GAE g^−1^ extract	Mahmoudi et al., 2015
Colored grains (18)	5.04–13.94; 7.97–14.12; 4.15–14.33; 8.20–8.94 mg g^−1^ GAE DW in black, blue, yellow, and mixed grain color, respectively	Abdel-Aal et al., 2012
Two- and six-rows, hulled and hulless normal and waxy grains (6)	171–554 mg g^−1^ DW	Gamel and Abdel-Aal, 2012
Hulled and hulless cultivars (12)	4.81–6.76 mg GAE g^−1^ DW	Holtekjølen et al., 2011
Hulled and hulless cultivars (10)	0.25–0.67 mg g^−1^ DW^$^	Andersson et al., 2008
Cultivars (10)	0.25–0.49 mg GAE g^−1^ DW	Dvořáková et al., 2008
Black, blue, purple grains (127)	0.19–0.40 mg GAE g^−1^ DW; unhulled (0.27 g^−1^) > hulled (0.21 mg g^−1^); blue and purple (0.27 mg g^−1^)> black (0.21 mg g^−1^)	Kim et al., 2007
**Maize (*****Zea mays*** **L.)**		
Blue-grain (7)	10.10–13.47 mg GAE g^−1^ DW	Urias-Lugo et al., 2015
Waxy (49)	0.005–0.012 mg GAE g^−1^ DW	Harakotr et al., 2015
Landrace populations (33)	1.32–2.62 mg of GAE g^−1^ DW	González-Muñoz et al., 2013
Inbred and landraces (10)	5.23–10.53mg GAE g^−1^ DW	Žilić et al., 2012
Red and blue colored grains (9)	3.11–8.18 mg GAE g^−1^ DW	Montilla et al., 2011
Waxy and normal yellow grains (4)	0.23–3.88 mg GAE g^−1^ DW	Hu and Xu, 2011
Colored and white grains (18)	1.70–3.40 mg GAE g^−1^ DW	Lopez-Martinez et al., 2009
**Rice (*****Oryza sativa*** **L.)**		
Diverse accessions (20)	0.40–5.62 and 0.44–6.62 mg GAE g^−1^ DW in two seasons	Shao et al., 2014a
Black, red, and white grains (3)	0.31–1.57 mg GAE g^−1^ DW	Shao et al., 2014b
Black and white grains (15)	0.15–0.37 mg g^−1^ DW; greater variation in total soluble phenolics in black (0.17–0.37 mg g^−1^) than white (0.15–0.17 mg g^−1^) grains	Park et al., 2012
Black, red, and white grains (6)	1.40–11.87 mg GAE g^−1^ DW	Bordiga et al., 2014
Cultivars with pigmented and non-pigmented grains (11)	0.001–0.014 mg GAE g^−1^ bran; higher phenols in pigmented than non-pigmented; greater phenols in black-colored *indica* than black-colored *japonica*	Huang and Ng, 2012
Black and red grains (13)	Black grains: 3.37–6.65 mg g^−1^ FAE DW; Red grains: 0.79–6.91 mg g^−1^ FAE DW	Sompong et al., 2011
Black grains (12)	23.65–73.67 mg GAE g^−1^ DW	Zhang et al., 2010
Colored and white grains (21)	1.07–4.25 mg FAE g^−1^ DW	de Mira et al., 2009
Wild (11)	2.47–4.07 mg FAE g^−1^ DW	Qiu et al., 2009
White, red and black grains (481)	1.08–1.24 mg GAE g^−1^ DW; black grains (10.56 mg) > red (4.70 mg) > white (1.52 mg)	Shen et al., 2009
**Rye (*****Secale cereale L***.**)**		
Cultivars (10)	0.49–1.08 mg g^−1^ DW^$^	Nyström et al., 2008
**Sorghum (*****Sorghum bicolor*** **(L.) Conrad Moench)**		
Colored and white grains (381)	2–14 mg GAE g^−1^ DW; proanthocyanidins high in brown while 3-deoxyanthocyanidins in red grains	Rhodes et al., 2014
Colored and white grains (287)	1–38 mg GAE g^−1^ DW; accessions with pigmented seeds had higher phenols	Dykes et al., 2014
Lines and hybrids with black grains (8)	5–20 mg GAE g^−1^ DW	Dykes et al., 2013
**Wheat (*****Triticum aestivum*** **L.)**		
Black, purple, and white grains (4)	0.51–0.66 mg GAE g^−1^ DW	Li et al., 2015
Cultivars (23)	2.90–5.65 mg GAE g^−1^ bran DW	Narwal et al., 2014
Spelt (6)	0.51–1.26 mg GAE g^−1^ DW	Gawlik-Dziki et al., 2012
Hard and soft Canadian wheat cultivars (21)	Soluble and bound phenols, respectively, ranged from 0.11–0.15 and 0.80–1.07 mg g^−1^ DW	Ragaee et al., 2012
Colored grains (13)	120–177 mg FAE 100 g^−1^ DW; purple and blue grains had greater phenolic than red-grains	Eticha et al., 2011
Market class (51)	3.41–6.70 mg g^−1^ GAE DW	Verma et al., 2008
Spring and winter wheat, spelt, durum, einkorn, emmer (175)	durum, spring, and winter wheat (0.61–0.70 mg FAE g^−1^); emmer (0.78 mg g^−1^)>einkorn (0.61 mg g^−1^)>Spelt (0.57 mg g^−1^); 2–3.6-fold variation within each group; winter wheat had greater variability (0.33–1.17 mg g^−1^)	Li et al., 2008

*Original data on phenylpropanoid constituents given in papers cited here were converted and presented into mg g^-1^ dry weight*.

$ *total anthocyanin (not the anthocyanin compounds) value was given in the original literature*.

Maize germplasm showed about 2-fold variation in total phenolics, except studies by Lopez-Martinez et al. (2009) and Hu and Xu (2011), who noted 16–20-fold variations. High phenolic content was found among Mexican landrace germplasm and in waxy corn germplasm from China. Black and purple grain showed higher levels of phenolics compared to red grain, while white kernels contained the lowest levels of phenolics (Lopez-Martinez et al., 2009; Hu and Xu, 2011; Montilla et al., 2011; González-Muñoz et al., 2013). Ferulic acid was the major phenolic, whereas *p*-coumaric, *o*-coumaric, vanillic, vanillin, and protocatechuic acids were found in significant quantities (Lopez-Martinez et al., 2009; Hu and Xu, 2011; Montilla et al., 2011; Žilić et al., 2012; González-Muñoz et al., 2013).

Total phenolics in rice germplasm showed up to 15-fold variation. One study involved 20 diverse rice accessions containing red and white grain types (Shao et al., 2014a) and another study had 481 accessions with black, red and white grain types (Shen et al., 2009); these showed large variability in total phenolics. Black rice germplasm contained higher levels of phenolics than red and white grain types, while white grain germplasm had the lowest levels of phenolics. Red grain types had higher levels of phenolics than white grain types, but lower levels than black grain types (Shen et al., 2009; Huang and Ng, 2012; Park et al., 2012; Shao et al., 2014a,b). Ferulic, p-coumaric, and salicylic acids were the major phenolic compounds (de Mira et al., 2009; Huang and Ng, 2012; Park et al., 2012), with pigmented rice grain containing greater soluble phenolic compounds than non-pigmented rice grain.

Wide range variations (7–38-fold) for total phenols were noted among sorghum germplasm (668 accessions) differing in grain color, while eight lines and hybrids showed 4-fold differences in phenols. High levels of total phenols were observed in pigmented genotypes (Dykes et al., 2014). Grain color had a significant effect on phenolic compounds. For example, red grain contained higher amounts of 3-deoxyanthocyanidins than brown or white grain, while brown grain contained significantly greater mean proanthocyanidins than red, white, or yellow grain (Rhodes et al., 2014). The grouping of sorghum germplasm based on biological and geographical origin also significantly affected total phenols and phenolic compounds. For example, *bicolor* and *caudatum* germplasm as a group had higher mean total phenols than *durra* and *guinea*, while *bicolor* and *guinea-caudatum* had higher mean proanthocyanidins and *bicolor-durra* and *guinea-caudatum* contained higher mean 3-deoxyanthocyanidins (Rhodes et al., 2014).

Wheat landraces and cultivars showed narrow variations (1.3–2.5-fold) for mean total phenols. Ferulic and *p*-coumaric acids were the major phenolics, while other compounds such as vanillic, syringic, 2,4-dihydroxybenzoic, and sinapic acids were also reported (Siebenhandl-Ehn et al., 2007; Li et al., 2008; Gawlik-Dziki et al., 2012; Ragaee et al., 2012). Some cultivar groups showed larger variability than others. For example, Li et al. (2008) showed greater range variations in winter wheat (3.6-fold; 0.33–1.17 mg g^−1^) than other cultivar groups (1.8–2.3-fold; spring: 0.46–0.89 mg g^−1^; durum: 0.54–1.08 mg g^−1^; spelt: 0.38–0.73 mg g^−1^; einkorn: 0.45–0.82 mg g^−1^; emmer: 0.51–1.16 mg g^−1^). Canadian “Western Red” spring wheat cultivars also showed greater variability in total phenols, with 4.62–6.70 mg g^−1^ in bran (Verma et al., 2008). Ancient cultivars showed significantly more phenolic compounds and isomer forms than modern wheat cultivars (Dinelli et al., 2009). However, ancient cultivars differed only slightly from modern wheats for most of the bioactive compounds (Laus et al., 2015; Shewry and Hey, 2015).

The key to successful crop improvement is a continued supply of genetic diversity that includes new or improved variability for target traits. Research toward the identification of health-promoting germplasm (i.e., containing more phenylpropanoids) is still in its infancy. Between 2009 and 2015, only a set of 4214 germplasm accessions of cereals and legumes were evaluated (Tables 1–5). Clearly, more investment is needed to identify germplasm that is rich in phenylpropanoids; screening of representative sets, such as core (Frankel, 1984) and mini core (Upadhyaya and Ortiz, 2001) subsets, will facilitate this task.

## Developing elite germplasm/cultivars high in phenylpropanoids

Variation in seed color is associated with significant differences in phytochemicals. An indirect screening method that includes color parameters (i.e., L^*^, lightness; b^*^, yellowness; a^*^, redness; H^*^, hue angle) with an automatic color difference meter can be used for selecting phenylpropanoid-rich crops (Shen et al., 2009; Jaafar et al., 2013; Sharma et al., 2014).

### Legumes

Soybean seeds are highly nutritious because they are rich in protein (>40%), oil (>18%), soluble carbohydrates (15%), and dietary fiber (15%). These seeds are exceptionally rich in isoflavones, such as genestein, daidzein, and glycetein, which are among the major components. High isoflavone cultivars are available in most soybean growing areas. The *tofu* (large-seeded) and the *natto* types (small-seeded, eaten as whole seeds) are grown in the USA and Canada (Anderson and Wolf, 1995). Soybean mutants modify seed chemistry based on single gene traits or by showing strong additive effects (Boerma and Specht, 2004). Combining genes to achieve the desired genotypes and capitalize on epistatic effects is being undertaken to improve this crop, both as food and feed (Wilson, 2012). Mutants enable genetic flexibility in tailoring soybean seed composition. The negative correlation between grain protein and isoflavone concentration might impose some biological constraint on the selection for these two beneficial traits (Murphy et al., 2009b). In contrast, similar relationships between isoflavone and linolenic (18:3) will be beneficial to the development of cultivars with high isoflavone and lower linolenic acid, the most undesirable trait (Wilson, 2012). The investment for crossbreeding soybean is smaller than that for transgenic breeding of this crop (Kumar and Ablett, 2010). Crossbred soybean is used for the improvement of transgenic soybean, because the gene base for crossbreeding is much wider than for transgenic breeding (Carter et al., 2004; Cui et al., 2004; Kumar and Ablett, 2010). It will be a challenge to maintain grain yield competitive soybean cultivars in a conventional background, as compared to transgenic soybean. This is likely to remain as a novel food until other traits are added to the trait profile of new cultivars. Despite these challenges, new cultivars with dramatic improvements in food and nutraceutical traits will emerge from global breeding programs. Soybean genomics will improve its breeding, which will greatly benefit human diets (Schmutz et al., 2010).

### Cereals

Crossbreeding has led to cultivars and breeding populations rich in phenylpropanoids. For example, blue landraces in maize are a rich source of anthocyanins (Mendoza-Díaz et al., 2012). Highly productive blue maize hybrids adapted to subtropical environments or to Mexican highlands were bred with similar nutraceutical profiles to that of blue maize landraces (Urias-Peraldí et al., 2013; Urias-Lugo et al., 2015).

Some people prefer pigmented (red or black) rice grains due to their taste, texture, aroma, and ceremonial or medicinal value (Sweeney et al., 2006). Wickert et al. (2014) bred the cultivars “SCS119 Rubi” and “SCS120 Onix,” with red and black grains, respectively, for specialty rice markets in Brazil. These cultivars had the same protein, lipid, carbohydrate, mineral, and fiber content as white rice, but contained higher levels of anthocyanins and phenolics than their ancestor cultivars. The mean grain yields of these cultivars were only 78% of the best white grain control (9.8 t ha^−1^), which suggests that greater efforts are needed to raise the yield potential of such rice cultivars.

Red pericarp introgression lines (ILs) that originated from interspecific crosses showed several-fold differences in total phenolics, flavonoids, proanthocyanidins, and tannins in brown and milled rice fractions (Sharma et al., 2014). Furthermore, their yield and physiological grain traits were comparable to those of their respective recurrent parents. More recently, advanced breeding lines were evaluated for yield and phenylpropanoids in China. Several lines combined high phenolic, anthocyanin, and antioxidant capacities with high grain yield potential (Zhang et al., 2015).

Sorghum lines “Tx3362,” “ATx3363,” and “BTx3363” with black seed pericarp have been registered as elite lines that have very high levels of 3-deoxyanthocyanins. These lines can be used as seed parents to produce hybrids rich in 3-deoxyanthocyanins or as breeding lines to produce additional lines with these unique characteristics (Rooney et al., 2013a,b). The black-grained sorghum hybrid “ATx3363 × RTx3362” yielded 70–76% of red grain hybrids, but it contained exceptionally high levels of total phenols, tannins, and 3-deoxyanthocyanins (Rooney et al., 2013a). Although a set of six black-grained sorghum hybrids contained significantly higher concentrations of phenols, tannins and 3-deoxyanthocyanins across environments, the grain yield of the best hybrid (“A05029 × Tx3362”) was only 78% of the commercial white-grained hybrid (“ATx631 × RT- x- 436”; Hayes and Rooney, 2015). This low yield of black-grained hybrids showed that black grain is associated with some unknown factors that have a negative influence on grain yield.

The HEALTHGRAIN project in Europe clearly demonstrated substantial variations for bioactive compounds (e.g., alkylresorcinols, β-glucan, carotenoids, folates, phenolics, sterols, tocols), which are genetically determined, although environmental effects were also conspicuous (Ward et al., 2008; Shewry, 2009; Van der Kamp, 2012). Nevertheless it appears feasible to select for high levels of bioactive compounds, which will lead to a new generation of healthy cereals. Various genebank accessions, and advanced breeding lines and cultivars with blue or purple grain characteristics (e.g., “Amethyst,” “Indigo,” “Capo,” “Saturnus,” “Skorpion”) have been identified or crossbred in wheat (Zeven, 1991; Qualset et al., 2005; Zheng et al., 2009; Eticha et al., 2011; Guo et al., 2011; Jaafar et al., 2013; Martinek et al., 2014). There has been increased use of wheat genetic resources with different grain colors to develop blue- or purple-grain wheat cultivars (Martinek et al., 2014). Genetic research suggests that it is possible to increase the anthocyanin content using different genetic backgrounds for purple pericarp and blue-aleurone germplasm in wheat. The total anthocyanin content among breeding lines ranged from 0.018 to 0.298 mg g^−1^ (mean 0.13 mg g^−1^), and many lines contained anthocyanins in amounts ≥0.1 mg g^−1^ (Jaafar et al., 2013). The Crop Development Center at the University of Saskatchewan, Canada, bred the purple wheat cultivar “AnthoGrain™” that contained twice the anthocyanin content of earlier cultivars (http://www.agwest.sk.ca/blog/posts/wheat-of-the-future-might-be-purple.html). More recently, Gordeeva et al. (2015) bred near isogenic wheat lines (NILs) carrying various combinations of purple pericarp (*Pp*) alleles, using marker-aided back-crossing. These NILs represent a useful resource for studying the effects of grain pigmentation on other wheat traits.

A marker-aided recurrent backcrossing scheme using a high anthocyanin tropical maize line carrying regulatory genes (i.e., *B1, Pl1*) that are associated with anthocyanin production successfully converted traditional yellow popcorn into anthocyanin-rich popcorn (Lago et al., 2013, 2014). The popping ability, the expansion capacity of the kernel, the flake volume, and the taste preference of the bred cultivar were similar to the commercial yellow popcorn line.

## Accessing consumer behavior to eating foods high in phenylpropanoids

Consumer acceptance of phytochemicals (such a flavonoids and phenols) in foods is widely recognized as a key factor for market orientation of new products. Bornkessel et al. (2011) noted that acceptance is mainly influenced by three factors: consumer characteristics, their purchasing power, and the product characteristics. Consumer characteristics concern personal health status and consumer awareness of phytochemicals as a special ingredient associated with certain additional benefits. The three main focus areas include consumer acceptance in general, health aspects and ingredients. The acceptance is determined by various factors such as primary health concerns, consumer familiarity with the new food concept and with the functional ingredients, nature of the product carrier, and communication and publicity of the health benefits. New product acceptance could be hindered because of the taste even though consumers perceive the benefits (Saba et al., 2010). Consumer knowledge of the health benefits of newly developed functional ingredients from phytochemicals appears to be relatively limited. Recent studies have shown that consumers of today are genuinely interested in products that are beneficial to their health. Middle-aged and elderly consumers tend to be substantially more health conscious than the younger generations. This is because they or members of their immediate social environment are much more likely to be diagnosed with a lifestyle-related disease (Nocella and Kennedy, 2012; Lähteenmäki, 2013).

The regulation of policies on health claims receives much attention worldwide because this helps consumers make informed healthy choices. Dean et al. (2007) researched public perceptions relating to different healthy grain-based foods (i.e., bread, pasta, biscuits) in Europe and how gender, nationality, type of food, health claims, and perceptions of the manufacturing processes influenced them. The results confirmed that perception of phytochemicals supplied by foods varies with gender, country, and differences in consumer perception of benefits relating to functional grain products. Men perceived more benefit in products with specific health claims than women did for products with general health claims. At the individual level, male level of perceived benefit in products with general health claims was, however, equally high as that of the women. Furthermore, modification of staple foods was perceived as being more beneficial than fun foods, and people preferred processes such as fortification and traditional cross-breeding to genetic modification. Lampila et al. (2009) investigated consumer perceptions of flavonoids using focus-group discussions in Europe. They noted that the average consumer was not familiar with the term “flavonoid.” However, consumer showed positive attitudes once informed about the beneficial effects of flavonoids on human health.

Consumer knowledge on the health effects of flavonoids is limited. Hence, there is need to improve marketing programs by including reliable nutritional information in food labels. The rising demand for such foods can be explained by the increasing cost of healthcare, the steady increase in life expectancy, and because older people wish to lead improved life quality during their “golden years” (Roberfroid, 2002; Siro et al., 2008). It should be noted that many industry-designed marketed nutritional supplements do not have beneficial effects for arresting the development of chronic diseases (such as cancer, cardiovascular diseases, diabetes, hypertension, inflammation, and obesity). This is partially due to the health effects resulting from a complex synergetic action of numerous phytochemicals supplied by foods or diets at nutritional doses and considering that a food is not a drug (Fardet and Rock, 2014).

## Outlook

Consumers worldwide are becoming aware of the benefits of nutraceutical foods. A paradigm shift is gradually emerging for the development of nutritionally dense cultivars in addition to integrating genes for productivity and stress tolerance. As a result, nutrient-dense cultivars are being bred in cereals and legumes. Breeding for staple crops rich in phenylpropanoids is just beginning. A positive development is the search for phenylpropanoid-rich germplasm both in cereals and legumes that show broad variations in genebank germplasm. Many breeding programs are transferring this variation into the cultigen pool. As expected some yield penalty has been noted, which should be further investigated to assess cause-effect relationships to overcome the negative trade-off using cross-breeding or biotechnology facilitated genetic betterment. A systematic evaluation of germplasm using representative subsets is the ideal approach to discover new sources of variations for these compounds. An approach combining high throughput assays and wet chemistry should be integrated to support such breeding programs. The sensory attributes and consumer acceptance of flavonoid-rich staple foods should be further investigated for their wide acceptance. A food matrix-based approach instead of a reductionist approach is suggested with investigations into the effects of these compounds on human health.

## Author contributions

SD and RO conceptualized the idea and finalized the outlines together with coauthors who contributed equally in manuscript writing and reading the full draft. RO, SD, and KS edited, while SD organized the paper.

### Conflict of interest statement

The authors declare that the research was conducted in the absence of any commercial or financial relationships that could be construed as a potential conflict of interest.
