# Supplementary material for: Exploiting Phenylpropanoid Derivatives to Enhance the Nutraceutical Values of Cereals and Legumes
Source: Front Plant Sci. 2016 Jun 3;7:763. doi: 10.3389/fpls.2016.00763 (PMC4891577; doi:10.3389/fpls.2016.00763)
Supplement: Supplementary file 1 [file Table1.pdf]

Supplementary Table 1. Abiotic and biotic factors impacting accumulation of phenylpropanoid constituent's in staple cereals and food legumes.

Crop	Factor	Compound	References
		Abiotic stress	
**Legumes**			
Common bean (*Phaseolus vulgaris* L.)	Drought	Polyphenols	Ovando-Martínez et al., 2014
Soybean (*Gylcine max* (L.) Merr)	UV-B, drought, CO_2, temperature	Isoflavone	Caldwell et al., 2005; Kim et al., 2011; Carrera and Dardanelli, 2015
Pea (*Pisum sativum* L.)	Drought	Total Phenolic	Juzoń et al., 2013
Mungbean (*Vigna radiata* (L.) R. (Wilczek)	Drought, ozone	Total Phenolic	Afzal et al., 2014; Chaudhary and Agrawal, 2015
**Cereals**			
Maize (*Zea mays* L.)	Drought	Flavonoids and phenolic	Ali et al., 2010
Barley (*Hordeum vulgare* L.)	Drought, salinity	Total phenolic	Ahmed et al., 2013a
Rice (*Oryza sativa* L.)	Salinity, CO_2, γ-irradiation, ozone	Phenolic, anthocyanins, and proanthocyanins	Chunthaburee et al., 2015; Wang and Frei, 2011; Goufo et al., 2014; Zhu et al., 2010
Wheat (*Triticum* spp.)	Drought, nitrogen, UV	Total phenolic acids, free phenolic acids, flavonoids, and lutein	Chakraborty and Pradhan, 2012; Lukow et al., 2012; Stumpf et al., 2015
		Biotic Stress	
**Legumes**			
Cowpea (*Vigna unguiculata* (L.) Walp.); chickpea (*Cicer arietinum* L.; soybean,	Postharvest pests	Phenolic acids	Sharma and Thakur, 2014
Common bean, cowpea, and soybean	Pathogens, fungus, bacteria & insects	Flavonoids and isoflavonoids	Treutte, 2006; Rubiales et al., 2015
Soybean	Host-plant defense	Isoflavonoids	Mapope and Dakora, 2013
**Cereals**			
Wheat (*Triticum aestivum* L.)	*Fusarium* species	Phenolic acids	McKeehen et al., 1999
Barley (*Hordeum vulgare* L.)	*Fusarium* species	Phenolic acids	Eggert et al., 2010
Maize (*Zea mays* L.)	Postharvest pests	Phenolic acids	Ahmed et al., 2013b; García-Lara and Bergvinson, 2014
Rice (*Oryza sativa* L.)	Postharvest pests	Phenolic acids	Bamisile et al., 2014
