# Supplementary material for: Exploiting Phenylpropanoid Derivatives to Enhance the Nutraceutical Values of Cereals and Legumes
Source: Front Plant Sci. 2016 Jun 3;7:763. doi: 10.3389/fpls.2016.00763 (PMC4891577; doi:10.3389/fpls.2016.00763)
Supplement: Supplementary file 2 [file Table2.pdf]

Supplementary Table 2. Transcriptional factors that regulate the genes involved in flavonoid biosynthesis.

Species	Genes	Transcription factors/ complex	Function	References
Thale Cress *(Arabidopsis thaliana L.)*	*CHS, CHI, F3H*	MYB11/ MYB12/ MYB111	Induces proanthocyanidin biosynthesis in seedlings	Stracke et al., 2007
	DFR, LDOX, UGT	PAP1/PAP2/MYB113/MYB114 (R2R3-MYBs)<br>TT8/GL3/EGL3 (bHLH)<br>TTG1 (WDR)	Induces anthocyanin biosynthesis in seedlings	Gonzalez et al., 2008
	F3H, DFR, LDOX	PAP1/PAP2/MYB113/MYB114<br>TT8/GL3/EGL3<br>TTG1<br>SPL9	Inhibits anthocyanin biosynthesis	Gou et al., 2011
	F3H, DFR, LDOX, UGT	R3-MYBs/CPC/MYBL2<br>TT8/GL3/EGL3	Inhibits anthocyanin biosynthesis	Dubos et al., 2008; Matsui et al., 2008; Zhu et al., 2009
Petunia (*Petunia* × *hybrida*)	*DFR, LDOX, UGT*	AN2/AN4 (R2R3-MYB)<br>AN1 (bHLH)<br>AN11 (WDR)	Activates anthocyanin biosynthesis in floral organs	Spelt et al., 2000
	DFR, LDOX, UGT	MYB27 (R2R3-MYB)<br>AN1	Inhibits anthocyanin biosynthesis	Albert et al., 2011
	DFR, LDOX, UGT	MYBx (R2R3-MYB)<br>AN1	Inhibits anthocyanin biosynthesis	Albert et al., 2011
Tobacco (*Nicotiana tabacum* L.)	*DFR, LDOX, UGT*	NtAN2 (R2R3-MYB)<br>NtAN1 (bHLH)	Activates anthocyanin biosynthesis in flowers	Bai et al., 2011; Pattanaik et al., 2010
Snapdragon (*Antirrhinum majus* L.)	*DFR, LDOX, UGT*	ROSEA1/ ROSEA2/ VENOSA (R2R3-MYB)<br>DELILA/MUTABILIS (bHLH)	Regulates anthocyanin biosynthesis in flowers	Schwinn et al., 2006
Maize (*Zea mays* L.)	*DFR, LDOX, UGT*	C1/PL (R2R3-MYB)<br>R/B (bHLH)<br>PAC1 (WD40)	Activates anthocyanin biosynthesis	Carey et al., 2004; Grotewold et al., 2000

Apple (*Malus domestica* Brokh. 1983)	*DFR*, *LDOX*, *UGT*	MdMYB1/MdMYB10/MdMYBA	Regulates anthocyanin biosynthesis in fruits	Ban et al., 2007; Espley et al., 2007
		MdbHLH3/ MdbHLH33		
Grape (*Vitis vinifera* L.)	*LAR1*, *ANR*	VvMYBA1/2/5a/5b (R2R3-MYBs)	Activates anthocyanin biosynthesis in skin and seeds	Deluc et al., 2006; Matus et al., 2010; Hichri et al., 2010
		VvMYC1/VvMYCA1 (bHLH)		
		VvWDR1/VvWDR2 (WD40)		
Strawberry (*Fragaria* × *ananassa* Duchesne)	*DFR*, *LDOX*, *UGT*	FaMYB10 (R2R3-MYB)	Activates anthocyanin biosynthesis	Lin-Wang et al., 2010
	DFR, *LDOX*, *UGT*	FaMYB1 (R2R3-MYB)	Represses anthocyanin biosynthesis	
Rice (*Oryza sativa* L.)	*DFR*, *ANS*	OsC1-MYB<br>Rc/Rd (bHLH)	Activates anthocyanin biosynthesis in plant shoot	Himi and Taketa, 2015; Hichri et al., 2011
Wheat (*Triticum aestivum* L.)	*DFR*	TaC1-A1/ B1/D1	Regulates anthocyanin synthesis in vegetative tissues	Himi and Taketa, 2015
Barley (*Hordeum vulgare* L.)	*DFR*	Hvmyb10<br>HvbHLH1<br>HvC1	Regulates proanthocyanidin biosynthesis is grains	Himi and Taketa, 2015
